# Cross-Condition Fault Diagnosis of Crane Slewing Bearings Based on a Lightweight Domain-Adaptive Graph Convolutional Network

**DOI:** 10.3390/s26175433

**Published:** 2026-08-27

**Authors:** Wuben Yang, Qiangyin Wu, Anding Wu, Lipeng Su, Yifan Lou, Jiafu Wu, Zhaoyi Wang, Cancan Yi

**Affiliations:** 1 Wenzhou Special Equipment Inspection & Science Research Institute, Wenzhou 325000, China; 2 Key Laboratory of Metallurgical Equipment and Control Technology, Ministry of Education, Wuhan University of Science and Technology, Wuhan 430080, China

**Keywords:** crane slewing bearing, cross-condition fault diagnosis, graph convolutional network, domain adaptation, semi-supervised learning

## Abstract

Cross-condition fault identification for crane slewing bearings is difficult because low rotational speed, heavy loading, and operating-condition variation jointly weaken fault-induced impulses and alter the distribution of monitoring data. In addition, vibration and acoustic emission signals describe different aspects of bearing degradation, making fixed multi-sensor fusion insufficient when sensor sensitivity changes across fault states. This study proposes a Lightweight Domain-Adaptive Graph Convolutional Network (LDAGCN) for cross-condition diagnosis. The model uses six vibration channels and one acoustic emission channel as synchronized heterogeneous inputs. Two compact one-dimensional encoders first learn modality-specific temporal representations, after which a feature-wise gate determines the relative contribution of each sensing modality. The fused embeddings in every mini-batch are regarded as graph nodes, and a sparse sample graph is reconstructed from Top-*k* cosine similarities. Multi-receptive-field graph convolution then aggregates one-hop and higher-order neighborhood information, while a residual connection limits excessive modification of the original fused features. To reduce the discrepancy between operating conditions, the training objective combines source-domain classification, adversarial domain discrimination, maximum mean discrepancy, and supervision from a small labeled target-domain adaptation subset. Experiments were carried out on a dedicated crane slewing-bearing test rig containing normal, inner-race fault, outer-race fault, and B1 localized-fault states. On the target-condition test set, LDAGCN achieved an accuracy of 97.22%, a Macro-F1 score of 97.21%, a Macro-Precision of 97.37%, and a Macro-Recall of 97.22%. The proposed model also outperformed 1D-CNN, ResNet1D, CNN-LSTM, DANN, MMD-DAN, and DeepCORAL under the same data partition. The confusion matrix and t-SNE projection indicate that the learned representation reduces the source–target distribution gap while retaining fault-class separation. The ablation results further indicate that the acoustic emission information, gated fusion, graph-based association learning, and domain adaptation complement each other in terms of the final diagnostic performance, while maintaining a lightweight architecture that is suitable for practical monitoring.

## 1. Introduction

Unlike conventional rolling bearings, crane slewing bearings rotate at relatively low speeds while simultaneously carrying axial forces, radial forces, and overturning moments. Their measured responses are therefore influenced not only by local defects but also by structural vibration, load variation, repeated impact, and sensor location. Under such conditions, fault-induced impulses are often weak and strongly nonstationary. Moreover, changes in rotational speed or applied load can alter the amplitudes, occurrence intervals, and spectral-energy patterns associated with the same defect. As a result, class boundaries learned from one operating condition may no longer remain valid under another condition, and responses produced by different fault types may partially overlap in the target condition. The principal difficulty addressed in this study is therefore to preserve fault-related discrimination when the data distribution changes between operating conditions.

Data-driven fault diagnosis has gradually moved from manually designed indicators toward representations learned directly from monitoring signals. Das et al. and Chen et al. reviewed the development of machine learning and deep transfer learning methods for rotating machinery and bearing diagnosis [1,2]. From the perspectives of domain adaptation and industrial deployment, existing surveys have further summarized statistical matching, adversarial learning, and transferable representation learning under varying operating conditions [3,4]. Chen et al. discussed the design principles and future directions of transfer learning-based intelligent diagnosis in automation systems [5]. These studies confirm that knowledge obtained from a labeled operating condition can reduce dependence on target-condition annotations. Nevertheless, most available benchmarks concern conventional rolling bearings tested under relatively controlled laboratory environments. Low-speed and heavy-load slewing bearings have received less attention, although their weak fault responses and fluctuating loads create a more pronounced cross-condition diagnostic challenge. Multi-sensor fusion provides an effective means of extending fault-information coverage. Recent reviews have summarized commonly used sensor combinations, fusion levels, and practical challenges in rotating-machinery diagnosis [6,7]. CNN-based fusion and sensor-coupling methods have shown that measurements from different channels can compensate for incomplete single-sensor information [8,9]. Attention-guided fusion and multi-branch residual learning further allow sensor-specific features to be extracted before cross-channel integration [10,11]. Hybrid multimodal fusion and audio–vibration joint diagnosis have also been investigated to improve robustness under complex interference [12,13]. More recent studies have incorporated adaptive signal decomposition, multi-attention mechanisms, and interpretable multi-sensor representations into fault diagnosis [14,15]. In low-speed and heavy-load bearings, acoustic emission measurements are particularly useful for capturing weak, localized, and high-frequency transient events [16]. However, many existing approaches still rely on direct concatenation, fixed coefficients, or globally shared attention weights, and their effectiveness under a pronounced source–target distribution shift remains insufficiently studied.

Cross-condition diagnosis is usually addressed by transferring knowledge from a labeled source condition to a different target condition. Subdomain matching and enhanced transfer networks reduce distribution discrepancies through class-related statistical constraints or transferable feature combinations [17,18], while adversarial transfer learning and mechanism-assisted multi-domain adaptation seek to suppress condition-specific information while retaining fault-discriminative characteristics [19,20]. However, global marginal alignment does not necessarily preserve class-wise correspondence, because samples from different fault categories may still be mapped to adjacent feature regions. Moreover, most unsupervised approaches ignore the limited target-condition labels that can be obtained through equipment inspection or expert verification. These labels can provide category anchors on the target side and thus are practically useful and very realistic in practice.

Graph-based diagnosis introduces relational information that cannot be represented when each signal window is processed independently. Multi-receptive-field graph convolution and knowledge-informed graph construction have demonstrated the value of aggregating information from related samples or system components [21,22]. Domain-adversarial sample graphs and multi-graph networks further incorporate relational structure into cross-condition feature transfer [23,24]. Dilated *K*-nearest-neighbor graphs and multi-sensor graph-attention models extend this idea by learning broader neighborhoods or heterogeneous sensing interactions [25,26]. Nevertheless, most existing diagnostic graphs are constructed from vibration-only representations. Relatively little attention has been paid to graphs formed from jointly learned vibration and acoustic emission features. Moreover, the computational complexity of some graph architectures limits their application in field monitoring systems. Existing studies indicate that graph propagation can enrich sample representations by incorporating relational information from similar observations. Nevertheless, most diagnostic graphs are constructed using vibration-only features, while the potential benefit of forming sample relationships from jointly learned vibration and acoustic-emission representations remains insufficiently investigated. In addition, complex graph architectures may introduce computational overhead that limits their use in on-site monitoring devices.

Despite the progress achieved in sensor fusion, domain adaptation, and graph-based diagnosis, their coordinated use for low-speed and heavy-load crane slewing bearings remains insufficiently explored. Three issues are particularly relevant to the present task. First, the contributions of vibration and acoustic emission information are commonly treated as fixed or globally shared. Second, distribution alignment is often performed without explicitly preserving the relational geometry of fault samples. Third, the computational burden of complex graph architectures restricts their use in on-site monitoring systems.

To address these gaps, this study proposes a Lightweight Domain-Adaptive Graph Convolutional Network (LDAGCN). The main contributions are summarized as follows:

(1) To address the insufficient use of complementary information from vibration and acoustic-emission signals under low-speed conditions, a dual-branch lightweight encoder with feature-wise gated fusion is developed. The proposed mechanism adaptively balances the contributions of the two sensing modalities and improves fault-related feature representation without introducing a large computational burden.

(2) To address the difficulty of preserving inter-sample relationships under operating-condition variation, a dynamic Top-k sample graph and multi-receptive-field graph-convolution module are introduced. The proposed graph representation captures sample relationships over different propagation ranges while retaining the original fused information through a residual connection.

(3) To reduce source–target distribution discrepancy while preserving target-side class discrimination, a semi-supervised domain-adaptation strategy combining adversarial learning, MMD regularization, and limited labeled target samples is developed. This strategy improves cross-load diagnostic robustness under limited target-domain supervision.

## 2. Proposed LDAGCN Model

### 2.1. Overall Framework

**Figure 1:**
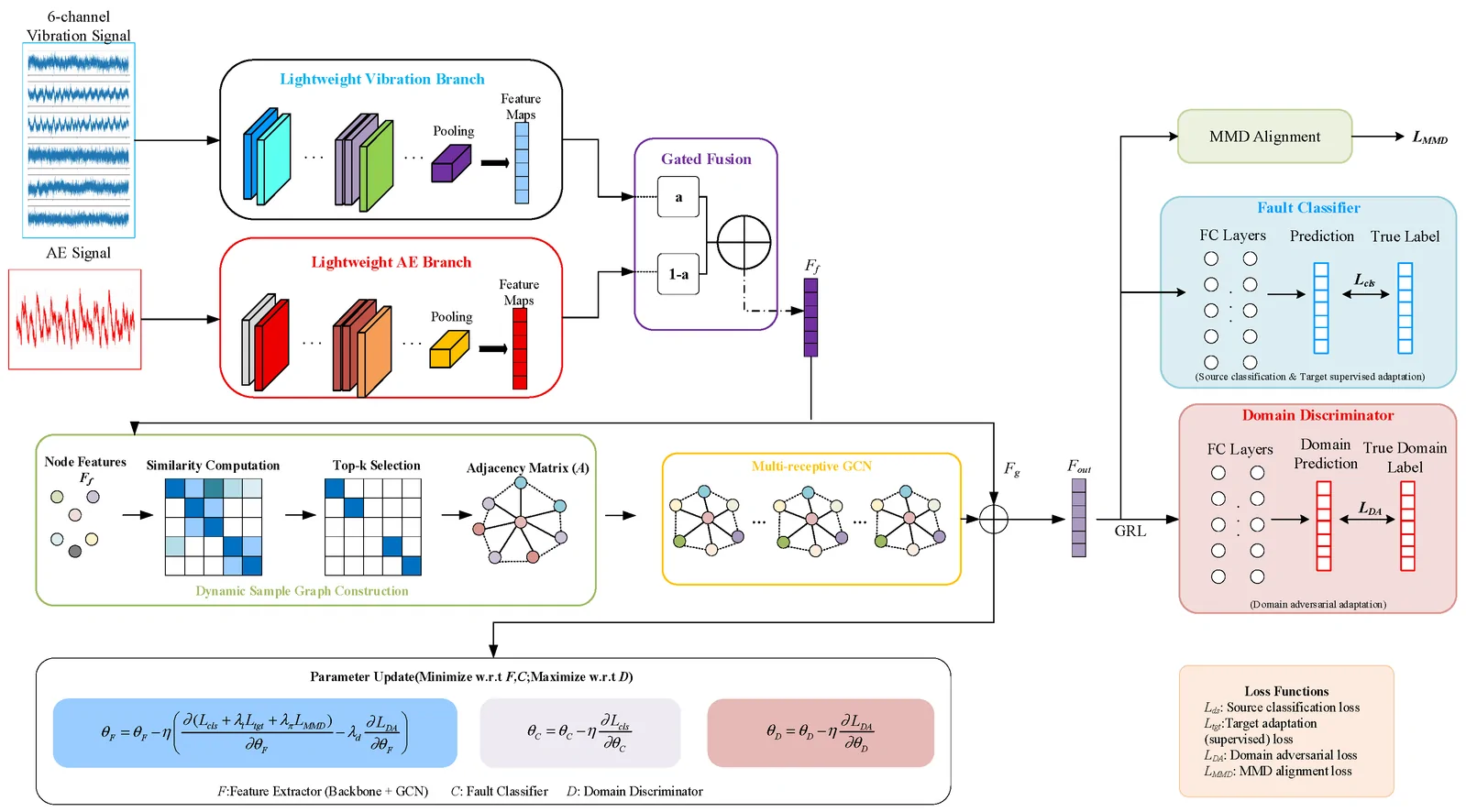
LDAGCN-driven model for cross-condition fault diagnosis of crane slewing bearings.

Figure 1 summarizes the information flow and training constraints of LDAGCN. The synchronized six-channel vibration windows and acoustic emission windows are first mapped into two modality-specific representations by independent lightweight encoders. A feature-wise gate then combines the two representations, and the resulting fused embeddings are used to update a Top-*k* sample graph for the current mini-batch. Multi-receptive-field graph propagation refines the node embeddings by incorporating information from directly connected and higher-order neighboring samples.

The refined representation is subsequently delivered to the fault classifier, which distinguishes the N, I, O, and B1 states. During training, the domain discriminator and the MMD term act on the high-level representation, while the fully labeled source samples and the limited labeled target adaptation samples provide class supervision. In this manner, sensor-specific encoding, cross-modal fusion, inter-sample relation learning, and operating-condition adaptation are optimized within a unified framework.

### 2.2. Multi-Sensor Lightweight Feature Extraction

For each signal window, the six synchronized vibration signals were stacked along the channel dimension to form a vibration input tensor $X_{v}\in {\mathbb{R}}^{6\times L}$, where *L* denotes the window length. The acoustic-emission signal was represented as $X_{a}\in {\mathbb{R}}^{1\times L}$. The six vibration channels were jointly processed by the vibration encoder, whereas the acoustic-emission signal was processed by an independent single-channel encoder. The outputs of the two branches were subsequently combined through the feature-wise gated fusion module.

Let the six-channel vibration input and the acoustic emission input be denoted by $X_{v}$ and $X_{a}$, respectively. A lightweight vibration branch $E_{v}(\cdot )$ and a lightweight acoustic emission branch $E_{a}(\cdot )$ are constructed to extract fault-related features from the two sensing modalities:

(1) $$F_{v}=E_{v}(X_{v})$$

(2) $$F_{a}=E_{a}(X_{a})$$

where $F_{v}$ and $F_{a}$ denote the vibration and acoustic emission features, respectively. Both branches consist of lightweight one-dimensional convolutional modules, normalization layers, nonlinear activation functions, and pooling layers. Compared with complex deep networks, the lightweight branches retain the ability to represent local impulsive characteristics and temporal patterns while reducing the number of parameters and computational cost.

Vibration signals generally reflect the global dynamic response of a mechanical structure, whereas acoustic emission signals are more sensitive to localized damage and transient impacts. Therefore, modeling the two types of signals separately using a dual-branch architecture helps preserve heterogeneous information from multiple sources and provides complementary representations for subsequent feature fusion.

### 2.3. Gated Fusion Module

The key to multi-sensor fusion lies in appropriately balancing the contributions of information from different sensors. Direct feature concatenation or fixed weighting may introduce excessive redundant information and cannot readily adapt to variations in sensor sensitivity across different fault conditions. To address this issue, a gated fusion module is designed to adaptively generate fusion weights from the vibration and acoustic emission features.

First, the two types of features are jointly mapped to obtain the gating coefficient:

(3) $$\alpha =\sigma (g([F_{v},F_{a}]))$$

where $\left[F_{v},F_{a}\right]$ denotes feature concatenation, g(.) represents the gating mapping function, and σ(.) is the Sigmoid function. The fused feature representation is given by

(4) $$F_{f}=\alpha F_{v}+(1-\alpha )F_{a}$$

where α represents the contribution of the vibration features, while (1−α) represents the contribution of the acoustic emission features. The gating coefficient α is automatically learned through backpropagation during model training. When α approaches 1, the corresponding feature dimension is dominated by the vibration features. When α approaches 0, it mainly depends on the acoustic emission features. When α is close to 0.5, the two sensing modalities make comparable contributions in that dimension. This mechanism enables the model to adaptively adjust sensor weights at the feature level, thereby yielding a more robust multi-sensor fused representation.

### 2.4. Dynamic Sample Graph Construction

To model latent relationships among windowed samples, LDAGCN dynamically constructs a sample-level graph from the fused representations of the current mini-batch. Let *B* denote the actual mini-batch size and $F_{f}\in {\mathbb{R}}^{B\times 64}$ denote the fused feature matrix, where each row represents one synchronized vibration–acoustic-emission window. Before graph construction, the fused features are projected into a 32-dimensional graph-embedding space:

(5) $$Z=P(F_{f})$$

where P(.) consists of two linear layers with a ReLU activation between them. The projected embeddings are then normalized along the feature dimension before sample similarity is calculated.

To further model the latent structural relationships among windowed samples, a dynamic sample graph is constructed based on the fused features $F_{f}$. Unlike a graph with a fixed topology, the dynamic sample graph does not rely on the spatial locations of sensors or the mechanical connectivity of the system. Instead, the windowed samples within each mini-batch are treated as graph nodes, while the feature similarities between samples are used to define the edge weights.

For the *i*-th and *j*-th samples, their similarity is defined as

(6) $$S_{ij}=\max\left(0,\frac{z_{i}^{T}z_{j}}{{∥z_{i}∥}_{2}{∥z_{j}∥}_{2}}\right)$$

where $z_{i}$ and $z_{j}$ denote the projected graph embeddings of samples *i* and *j*, respectively. Negative cosine similarities are truncated to zero before sparse neighbor selection.

(7) $$k_{B}=\min\{\max(k,1),B\}$$

The effective number of retained similarity entries is denoted by $k_{B}$. It is dynamically limited by the actual mini-batch size B, ensuring that the Top-*k* operation remains valid when the final mini-batch contains fewer samples than the nominal batch size.

(8) $$A_{ij}^{\mathrm{dir}}=\left\{\begin{array}{c} S_{ij},j\in {\mathrm{TopK}}_{k_{B}}(S_{i,:})\\ 0,\;\;\;\mathrm{otherwise} \end{array}\right.$$

The Top-*k* operation is independently applied to each row of the similarity matrix. Therefore, the resulting adjacency matrix $A^{\mathrm{dir}}$ is initially directed because the selection relation is not necessarily reciprocal. That is, sample *i* may select sample *j*, whereas sample *j* may not select sample *i*.

(9) $$A=\max\left[A^{\mathrm{dir}},{\left(A^{\mathrm{dir}}\right)}^{T}\right]$$

To obtain an undirected sample graph, element-wise maximum symmetrization is applied to the directed adjacency matrix. An edge is retained if either of the two corresponding samples selects the other as one of its high-similarity relations. Consequently, the adjacency matrix used for subsequent graph propagation is symmetric.

(10) $$\tilde{A}=A+I_{B}$$

(11) $$\hat{A}={\tilde{D}}^{-1/2}\tilde{A}{\tilde{D}}^{-1/2}$$

(12) $${\tilde{D}}_{ii}=\sum_{j=1}^{B}{\tilde{A}}_{ij}$$

Self-loops are added through the identity matrix $I_{B}$, allowing every node to retain its own representation during graph propagation. The self-loop also guarantees that each node has a nonzero degree even when its external similarity weights are weak or zero. Therefore, isolated zero-degree nodes do not occur in the normalized graph. Symmetric degree normalization is subsequently applied to obtain $\hat{A}$.

The neighborhood size *k* was treated as a validation hyperparameter rather than being manually fixed. Candidate values *k* ∈ {1, 2, 3, 4, 5, 8} were evaluated using the same data partition, random seed, batch size, learning rate, number of training epochs, and remaining model parameters. For each candidate value, model selection was performed according to the Macro-F1 score on the target-validation subset, without accessing the target-test labels. The optimal neighborhood size was determined as follows:

(13) $$k^{*}=\arg\underset{k\in K}{\max}\mathrm{MacroF}1_{\mathrm{val}}(k),\;k=\{1,2,3,4,5,6,7,8\}$$

**Figure 2:**
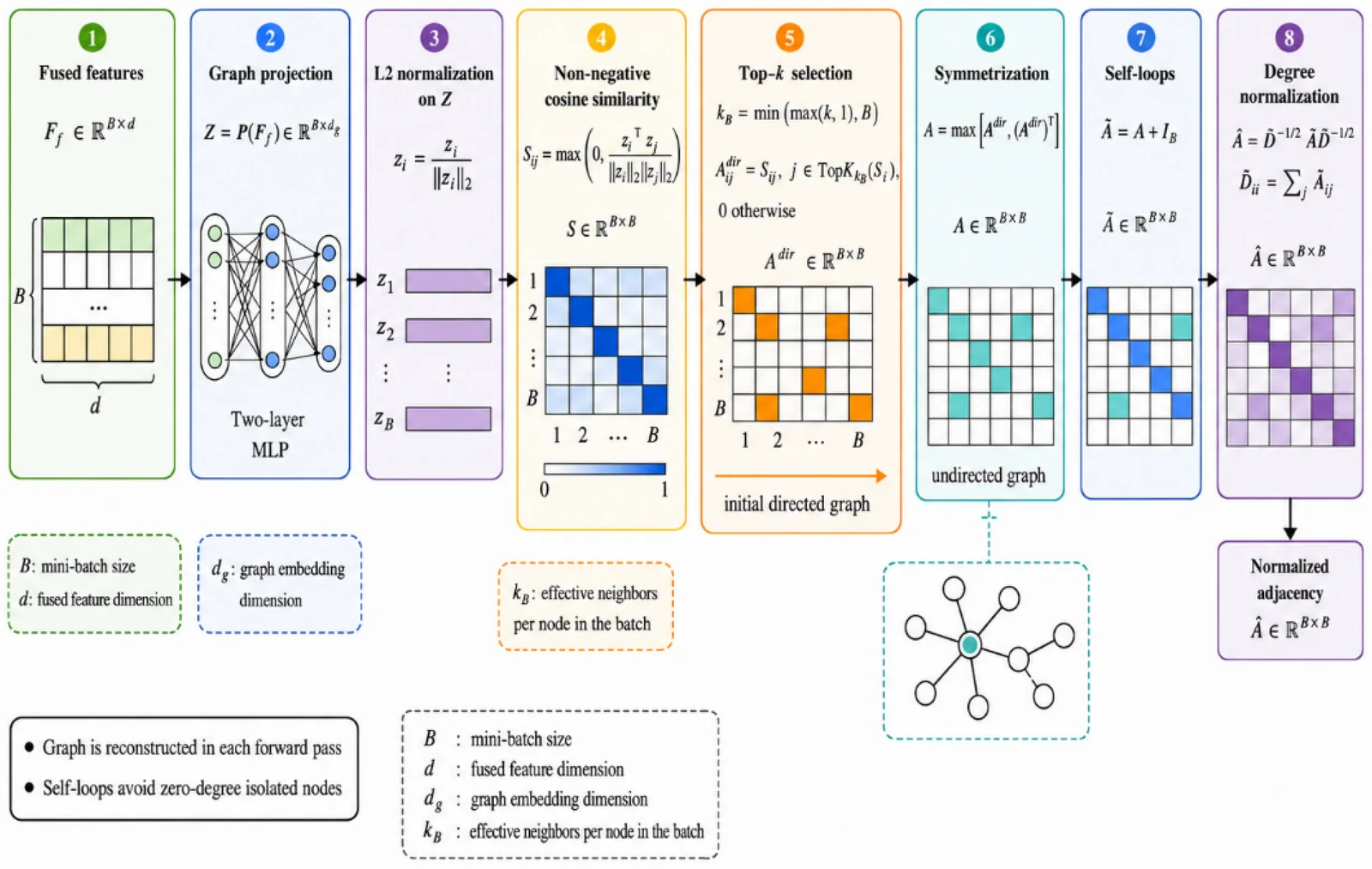
Dynamic mini-batch sample graph construction.

The neighborhood size *k* was treated as a validation hyperparameter and selected according to the Macro-F1 score on the target-validation subset without accessing the target-test labels. The complete dynamic graph-construction procedure described above is summarized in Figure 2, including feature projection, cosine-similarity calculation, Top-k neighbor selection, adjacency symmetrization, self-loop addition, and symmetric degree normalization.

### 2.5. Multi-Receptive-Field Graph Convolutional Feature Enhancement

**Figure 3:**
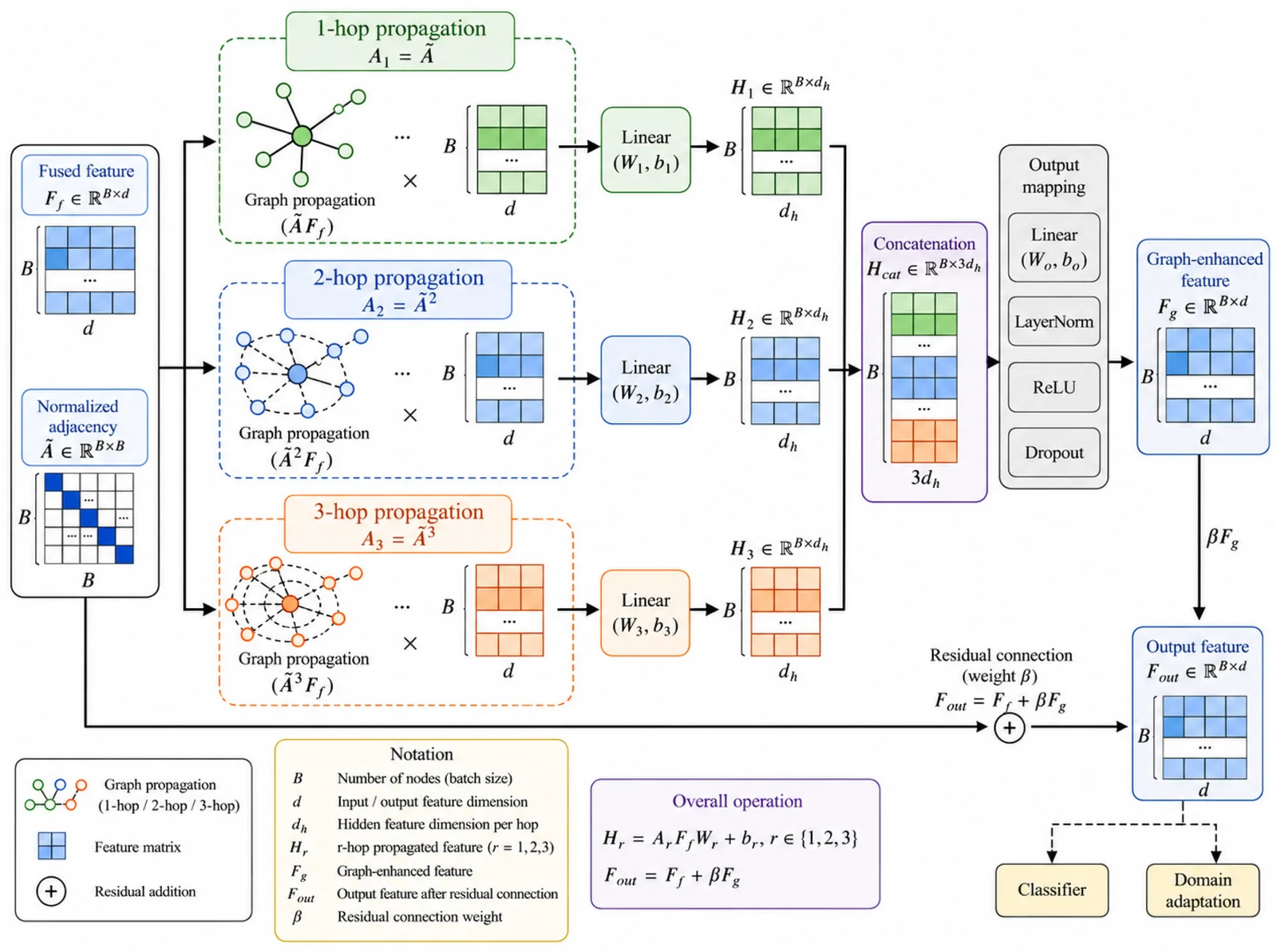
Multi-receptive-field graph convolutional feature enhancement.

After constructing the dynamic sample graph, a multi-receptive-field graph-convolution module is employed to propagate and aggregate node features at different neighborhood scales, as illustrated in Figure 3. Instead of sequentially stacking multiple conventional GCN layers, three parallel graph-propagation branches are constructed using different powers of the normalized adjacency matrix:

(14) $$A_{1}=\hat{A},\;A_{2}={\hat{A}}^{2},\;A_{3}={\hat{A}}^{3}$$

Here, $A_{1},A_{2}$ and $A_{3}$ represent the one-hop, two-hop, and three-hop propagation operators, respectively. For each receptive field, graph propagation and feature transformation are performed as

(15) $$H_{r}=A_{r}F_{f}W_{r}+b_{r},\;r\in \{1,2,3\}$$

where $F_{f}$ denotes the multimodal fused feature matrix. The weight matrices $W_{r}$ and bias terms $b_{r}$ are independently learned for the three propagation scales. Therefore, $H_{1},H_{2}$ and $H_{3}$ encode one-hop, two-hop, and three-hop inter-sample relationships, respectively.

The three graph representations are concatenated along the feature dimension:

(16) $$H_{\mathrm{cat}}=H_{1}∥H_{2}∥H_{3}$$

where ∥ denotes feature concatenation. The concatenated representation is then mapped back to the original feature dimension:

(17) $$F_{g}=\mathrm{Dropout}\left[\mathrm{ReLU}\left(\mathrm{LN}\left(H_{\mathrm{cat}}W_{o}+b_{o}\right)\right)\right]$$

where ReLU(.) denotes layer normalization, and $F_{g}$ is the graph-enhanced feature representation. Through the parallel propagation branches, each sample representation contains its own information and relational information aggregated from one-hop, two-hop, and three-hop neighborhoods.

To prevent the graph-convolutional output from excessively perturbing the original fused features, a residual enhancement strategy is adopted:

(18) $$F_{\mathrm{out}}=F_{f}+\beta F_{g}$$

where β is the graph residual coefficient, and $F_{\mathrm{out}}$ is the final high-level feature representation used for fault classification and domain adaptation. This residual design preserves the original vibration–acoustic-emission fusion information while incorporating structural relationships at different graph-neighborhood scales.

### 2.6. Domain-Adversarial Learning and MMD-Based Distribution Alignment

The graph-refined representation may still retain information associated with the operating condition. LDAGCN suppresses such condition-specific cues by combining a trainable domain classifier with an explicit distributional penalty. The domain classifier D(.) receives $F_{out}$ through a gradient reversal layer and predicts the binary domain label d. Its classification loss is written as

(19) $$L_{DA}=CE(D(F_{out}),d)$$

where d∈{0,1} identifies the source or target origin of a sample. During backpropagation, the domain classifier minimizes the domain-label error, whereas the gradient transmitted to the upstream feature extractor is multiplied by a negative coefficient. These opposite optimization directions cause the classifier to improve domain recognition while encouraging the feature extractor to remove information that separates the two operating conditions.

In parallel with the adversarial objective, the discrepancy between the empirical source and target feature distributions is constrained by MMD:

(20) $$L_{\mathrm{MMD}}={\left\|\frac{1}{n_{s}}\sum_{i=1}^{n_{s}}\phi (F_{s}^{i})-\frac{1}{n_{t}}\sum_{j=1}^{n_{t}}\phi (F_{t}^{j})\right\|}_{H}^{2}$$

where ϕ(.) denotes the kernel-induced feature mapping, and H represents a reproducing kernel Hilbert space. MMD alignment and domain-adversarial learning complement each other. The former encourages the source- and target-domain features to become statistically similar, whereas the latter promotes the learning of domain-indistinguishable representations from a discriminative adversarial perspective.

### 2.7. Semi-Supervised Adaptation Assisted by a Limited Number of Target-Domain Samples

Adversarial and MMD alignment reduce condition-related discrepancy at the domain level but do not directly determine the class boundaries of the target condition. A small labeled target adaptation subset is therefore introduced as a set of target-side class anchors. Its supervised loss is defined as

(21) $$L_{tgt}=CE(C(F_{t}^{l}),y_{t}^{l})$$

where C(.) denotes the fault classifier, $F_{t}^{l}$ represents the feature of the *i*-th labeled target-domain adaptation sample, and $y_{t}^{l}$ denotes its target-domain label. This loss provides the model with class-boundary information under the target operating condition, thereby improving the stability of target-domain fault diagnosis.

### 2.8. Overall Optimization Objective

Training is performed by minimizing the weighted combination of source classification, target adaptation supervision, adversarial domain learning, and MMD regularization:

(22) $$L_{\mathrm{total}}=L_{\mathrm{src}}+\lambda_{t}L_{\mathrm{tgt}}+\lambda_{d}L_{\mathrm{adv}}+\lambda_{m}L_{\mathrm{MMD}}$$

The overall training objective combines source-domain classification, limited target-domain supervision, domain-adversarial alignment, and MMD-based distribution alignment. Here, $L_{\mathrm{src}}$ denotes the source-domain classification loss, $L_{\mathrm{tgt}}$ corresponds to the supervised classification loss calculated using the labeled target adaptation samples, $L_{\mathrm{adv}}$ is the domain-adversarial loss, and $L_{\mathrm{MMD}}$ expresses the distribution discrepancy measured by maximum mean discrepancy. The coefficient $\lambda_{t}$ controls the contribution of target-domain supervision, $\lambda_{d}$ controls adversarial domain alignment, and $\lambda_{m}$ controls MMD-based statistical alignment. In the experiments, the corresponding coefficients were set to $\lambda_{t}$ = 0.15, $\lambda_{d}$ = 0.02, and $\lambda_{m}$ = 0.005, as summarized in Table 1.

## 3. Experimental Setup

### 3.1. Experimental Test Rig

**Figure 4:**
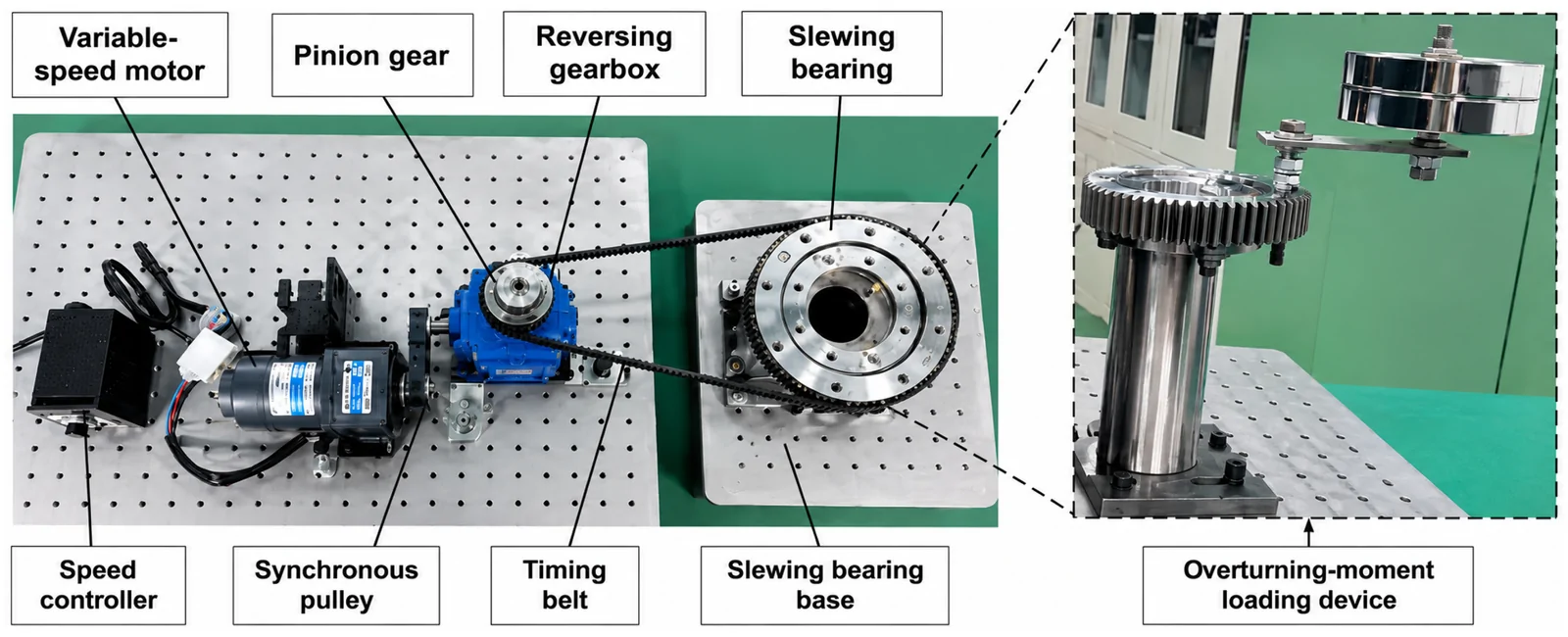
Experimental test rig for slewing-bearing fault diagnosis.

The experimental data were obtained from the crane slewing-bearing test facility shown in Figure 4. The facility integrates a slewing bearing, driving pinion, supporting base, transmission mechanism, variable-speed motor, controller, loading device, and multi-channel acquisition unit. Adjustments to the motor speed and applied load enable the bearing to operate under different mechanical conditions, thereby providing the condition-dependent datasets required in this study.

**Figure 5:**
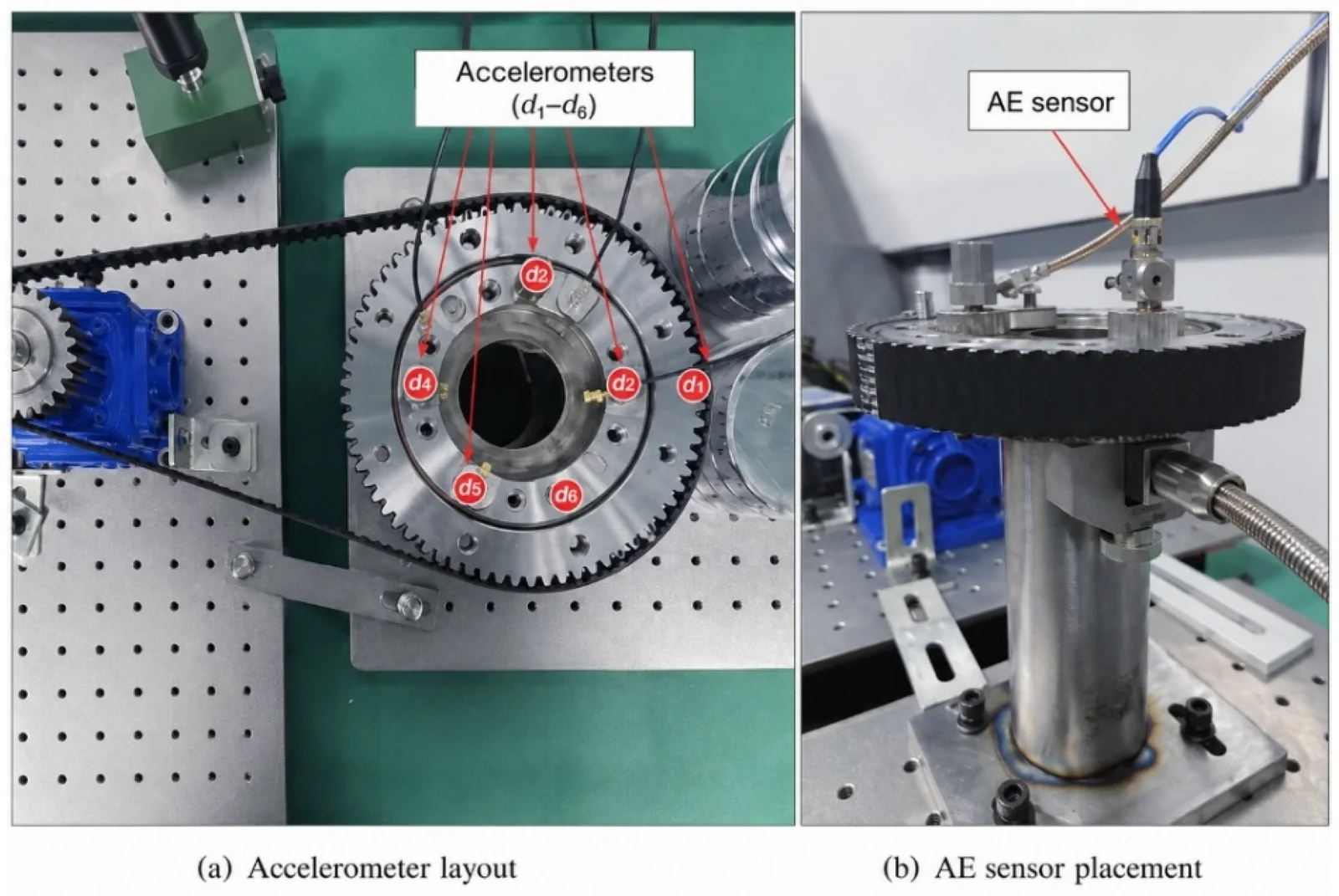
Sensor arrangement on the experimental test rig.

Seven synchronized sensing channels were used during each test. Six accelerometers were mounted at different positions around the slewing bearing to record spatially distributed vibration responses. An acoustic emission sensor was installed close to the damage-sensitive region to capture high-frequency transient activity associated with impacts, localized defects, and crack-related events. The installation positions of the vibration and acoustic emission sensors are presented in Figure 5.

Four bearing states were investigated: normal operation, inner-race fault, outer-race fault, and B1-type rolling-element or localized fault, denoted by N, I, O, and B1, respectively. Measurements obtained under the selected operating conditions were assigned to the source and target datasets. The source-condition samples provided the principal supervised information, whereas the target-condition samples were used for adaptation, validation, and final testing. Table 2 reports the structural parameters of the tested slewing bearing, and Table 3 defines the four diagnostic categories.

Three operating conditions were considered in this study. The slewing speed was fixed at 2 rpm, while the applied overturning force was set to 0 N, 30 N, and 60 N, respectively. Each condition contained four health states, including normal, inner-race fault, outer-race fault, and B1 fault. Five independent recordings were collected for each health state under each operating condition. To evaluate the cross-condition performance under different load variations, three source-to-target transfer tasks (T1, T2, and T3) were constructed. Task T1 used the 2 rpm–0 N condition as the source domain and the 2 rpm–30 N condition as the target domain. Task T2 transferred from 2 rpm–0 N to 2 rpm–60 N, while Task T3 transferred from 2 rpm–30 N to 2 rpm–60 N. These tasks represent different degrees of load variation at a constant rotational speed.

### 3.2. Fault Fabrication and Severity Description

**Figure 6:**
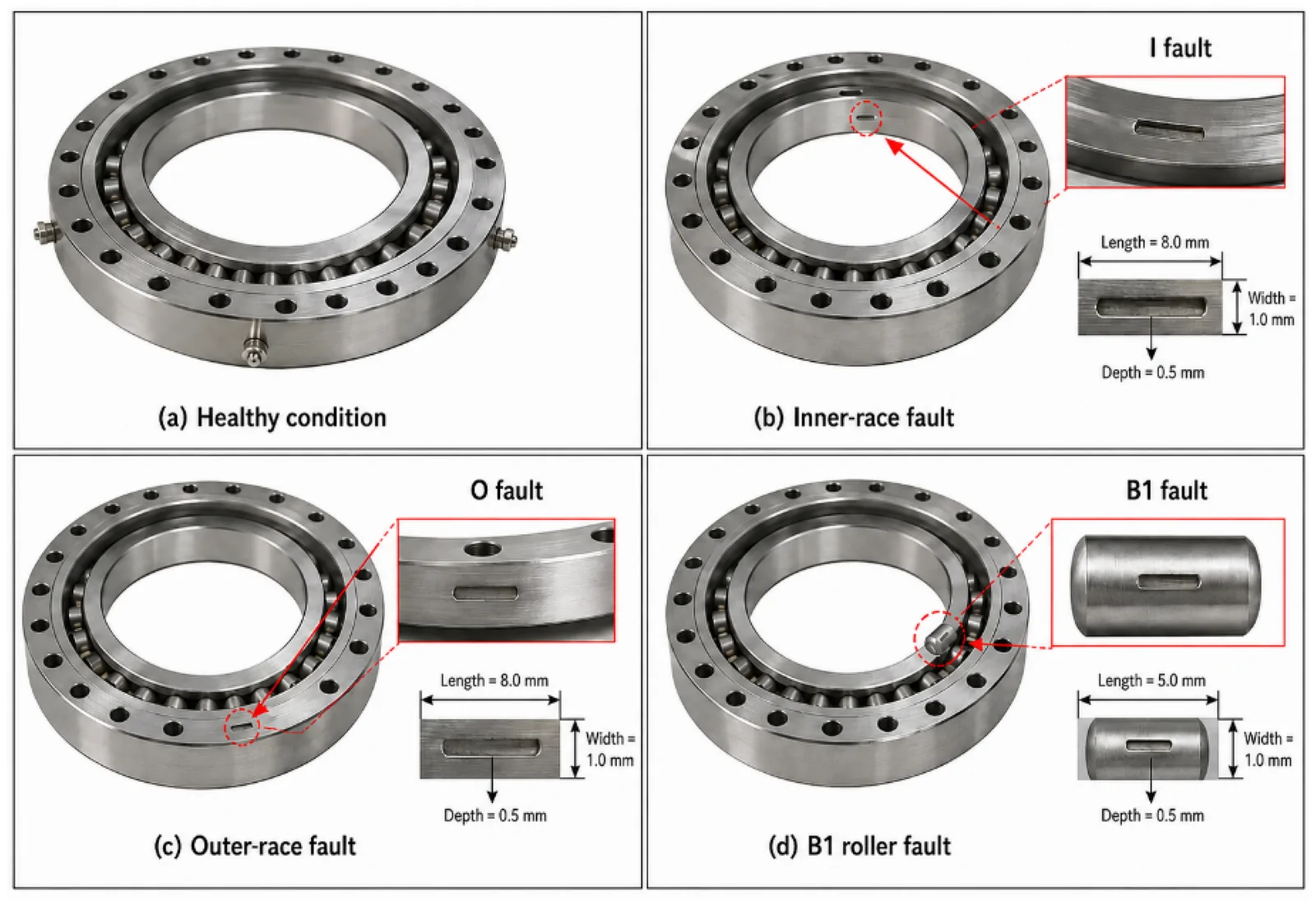
Schematic illustration of the healthy condition and the artificially seeded fault locations: (**a**) healthy condition; (**b**) inner-race notch; (**c**) outer-race notch; (**d**) B1 cylindrical-roller notch.

The I, O, and B1 faults used in this study were artificially seeded, localized defects rather than naturally degraded faults. Fault I was located in the inner raceway, Fault O was in the outer raceway, and Fault B1 was in the cylindrical roller. The defects were fabricated using electrical discharge machining (EDM). Their physical locations and nominal dimensions are summarized in Table 4. Figure 6 shows the healthy specimen and the locations of the artificially seeded inner-race, outer-race, and roller faults. The enlarged regions indicate the defect positions and nominal dimensions.

### 3.3. Signal Preprocessing and Window Segmentation

Both the original vibration and acoustic-emission signals are continuous time series. Directly feeding the full-length sequences into the model would increase the computational burden and may weaken the representation of localized fault-induced impulses. Therefore, a sliding-window strategy was employed to divide the synchronized signals into fixed-length samples. Let *L* and *S* denote the window length and step size, respectively. The *i*-th windowed sample is defined as follows:

(23) $$x_{i}=x[iS:iS+L]$$

**Figure 7:**
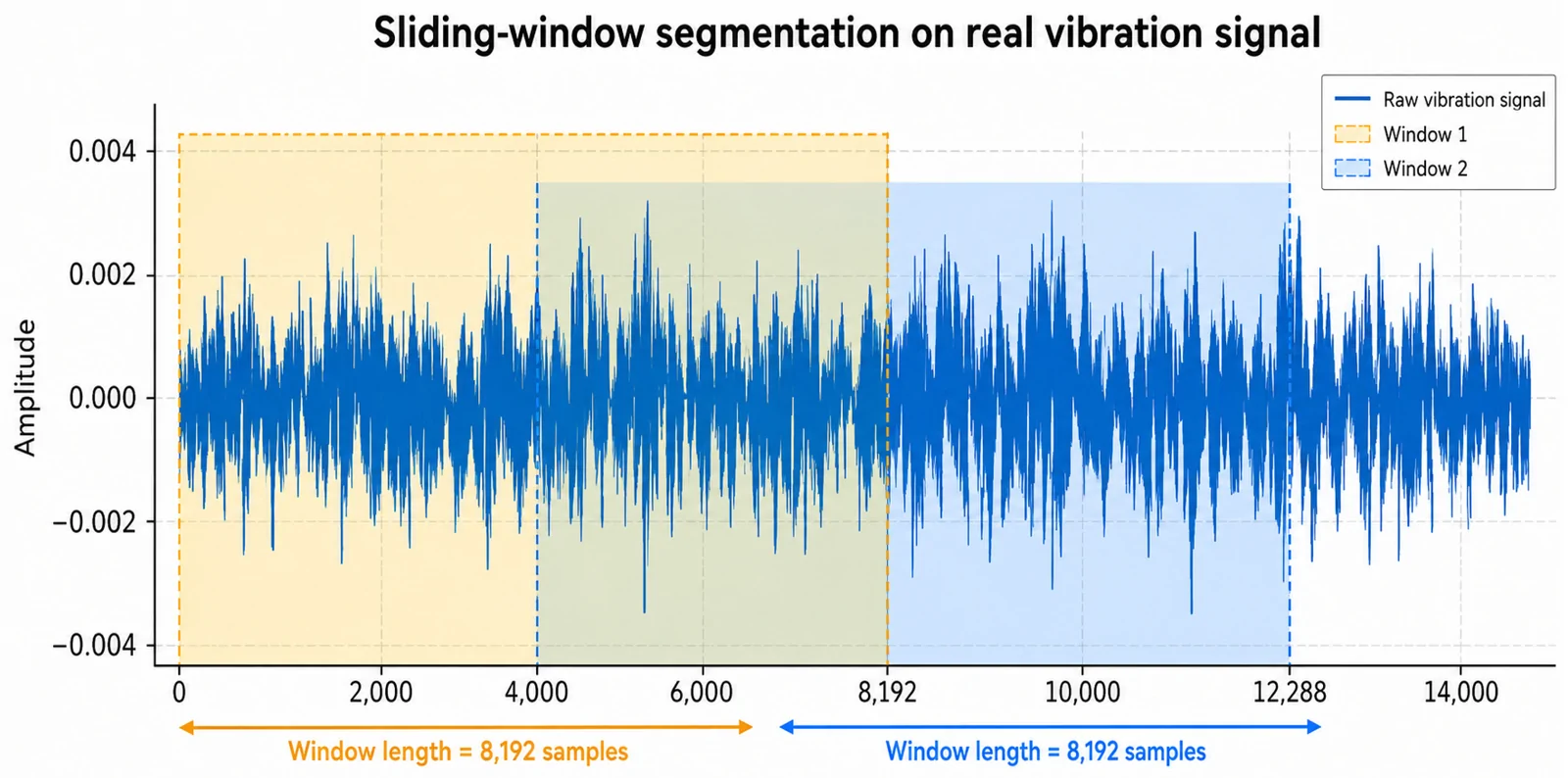
Schematic illustration of sliding-window segmentation.

Through sliding-window segmentation, each continuous signal is converted into a set of fixed-length windowed samples, as illustrated in Figure 7. This procedure preserves the local temporal structure while improving data utilization. In the experiments, the window length and step size were set to 8192 and 4096, respectively. Thus, adjacent windows partially overlap, increasing the number of available samples and improving the coverage of local fault-related features.

Since a sliding-window strategy with 50% overlap was adopted, special care was taken to avoid information leakage between different datasets. The source-domain training recordings and the target-domain adaptation, validation, and testing recordings were collected from independent continuous signals under different operating conditions. Sliding-window segmentation was then performed separately within each partition, ensuring that overlapping windows were confined to the same dataset and were never shared across different data partitions.

Each resulting window was subsequently normalized on a channel-by-channel basis. For each vibration or acoustic-emission channel, the mean and standard deviation were independently calculated within the current window, and z-score normalization was performed as

(24) $${\tilde{x}}_{c}(n)=\frac{x_{c}(n)-\mu_{c}}{\sigma_{c}+\varepsilon }$$

where $x_{c}(n)$ and ${\tilde{x}}_{c}(n)$ denote the original and normalized values of the *n*-th sampling point in channel *c*, respectively; $\mu_{c}$ and $\sigma_{c}$ are the mean and standard deviation of channel *c* within the current window; ε = 10^−8^ is introduced to avoid numerical division by zero. The normalization statistics were calculated independently for each channel and each window rather than using global dataset statistics. The same preprocessing procedure was applied to the source and target domains.

### 3.4. Source- and Target-Domain Partitioning

The cross-condition task was formed using measurements collected under two distinct operating conditions. Data from one condition served as the labeled source set, while data from the other condition were assigned to the target set. The target data were further separated according to their role in model development. A small labeled subset was used for target-side adaptation, a validation subset supported model selection and inference calibration, and the remaining samples were reserved for the final test. No target-test sample or label was accessed during parameter updating.

The source training set is represented by

(25) $$\left[D_{s}={\left(x_{s}^{i},y_{s}^{i}\right)}_{i=1}^{n_{s}}\right]$$

Similarly, the small labeled target-domain adaptation set is defined as

(26) $$\left[D_{t}^{l}={\left(x_{t}^{i},y_{t}^{i}\right)}_{i=1}^{n_{l}}\right]$$

The target-domain test set is expressed as

(27) $$\left[D_{t}^{test}=x_{t\;j=1}^{j\;n_{t}}\right]$$

where $x_{s}^{i}$ and $x_{t}^{i}$ denote the source- and target-domain samples, respectively, and $y_{s}^{i}$ and $y_{t}^{i}$ are their corresponding fault labels. The source and target domains share the same label space:

(28) $$\left[Y_{s}=Y_{t}=N,I,O,B1\right]$$

However, their feature distributions are different:

(29) $$\left[P_{s}(X)\neq P_{t}(X)\right]$$

**Figure 8:**
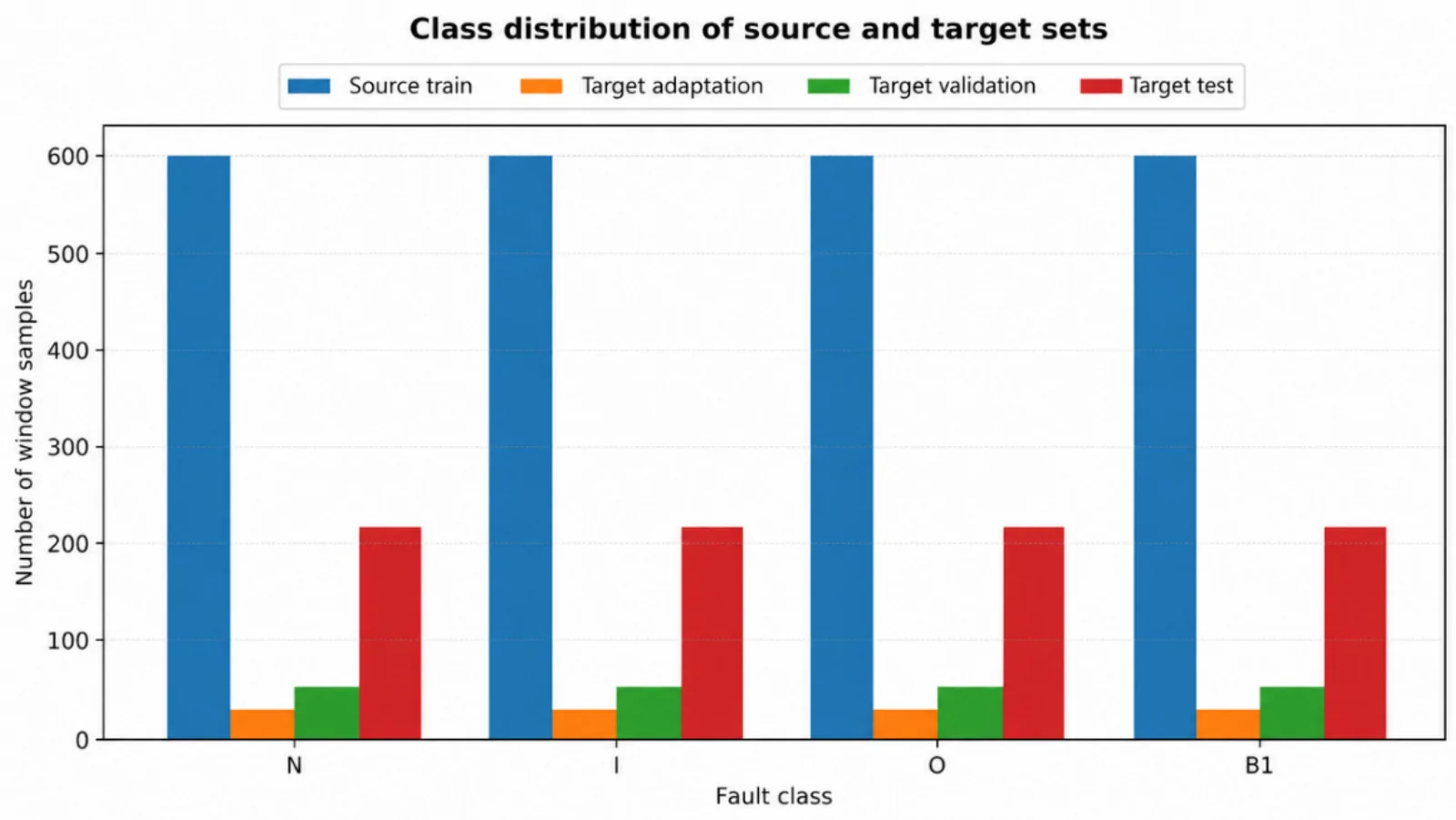
Sample distributions in the source and target domains.

The objective of this study is to learn a diagnostic model $\left(f:X_{t}\to Y_{t}\right)$ under the condition that the source domain is fully labeled while only a limited number of target-domain labels are available, such that accurate and stable fault identification can be achieved on the target-condition test samples. The sample distributions of the source and target domains are illustrated in Figure 8.

Figure 8 presents the class distributions of the source training set and the target adaptation, validation, and test sets. The source training set contained 600 samples per class. The target adaptation, validation, and test sets contained 30, 54, and 216 samples per class, respectively. Therefore, the four fault categories were equally represented in each dataset subset, and no class imbalance was observed. Consequently, no over-sampling or under-sampling strategy was applied during model training.

## 4. Comparative Experiments

To evaluate the effectiveness of the proposed LDAGCN, six representative approaches were selected as comparison models, including 1D-CNN [27], ResNet1D [28], CNN-LSTM [29], DANN [30,31], MMD-DAN [32], and DeepCORAL [33]. Among them, 1D-CNN, ResNet1D, and CNN-LSTM represent conventional deep feature extraction models, whereas DANN, MMD-DAN, and DeepCORAL represent typical domain adaptation methods. To ensure a fair comparison, all methods employed the same dataset partitioning, sliding-window segmentation, training protocol, testing settings, and evaluation protocol. Furthermore, to assess the robustness and reproducibility of different methods, all experiments were independently repeated five times using different random seeds (42, 52, 62, 72, and 82), and the reported results are presented as the mean ± standard deviation.

Performance was assessed using accuracy and macro-averaged precision, recall, and F1. Macro-F1, defined in Equation (30), was treated as the principal metric because it assigns equal importance to the N, I, O, and B1 classes.

(30) $$Macro-F1=\frac{1}{K}\sum_{k=1}^{K}F1_{k}$$

LDAGCN was optimized using Adam under the training configuration summarized in Table 5.

To ensure a fair comparison, all methods used the same synchronized multi-sensor input and data partitions. For each window, the six vibration signals and one acoustic-emission signal were provided to every comparison method. LDAGCN processed the six vibration channels and the acoustic-emission channel using two independent encoding branches. For the single-branch baseline models, including 1D-CNN, ResNet1D, CNN-LSTM, DANN, MMD-DAN, and DeepCORAL, the six vibration channels and one acoustic-emission channel were concatenated along the channel dimension to form a seven-channel input:

(31) $$X=X_{v}∥X_{a}\in {\mathbb{R}}^{7\times L}$$

where $X_{v}\in {\mathbb{R}}^{6\times L}$ denotes the six-channel vibration input, $X_{a}\in {\mathbb{R}}^{1\times L}$ denotes the acoustic-emission input, ∥ denotes channel-wise concatenation, and *L* denotes the signal window length. Accordingly, the input layer of each single-branch baseline was configured with seven input channels. All methods used the same preprocessing procedure; training, validation, and test partitions; and window length. Therefore, the comparison results were not influenced by differences in sensor-input information.

To ensure that the comparison results were not affected by insufficient baseline tuning, all baseline methods were optimized using a unified validation-based hyperparameter search. For 1D-CNN, ResNet1D, and CNN-LSTM, three learning rates and two weight-decay values were evaluated, resulting in six candidate configurations for each method. For DANN, MMD-DAN, and DeepCORAL, three learning rates and three adaptation-loss coefficients were evaluated, resulting in nine candidate configurations for each method. Therefore, a total of 45 candidate configurations were evaluated across the six baseline methods. The detailed search spaces, number of tuning trials, and selected configurations are reported in Table 6.

The optimal configuration of each baseline was selected according to the Macro-F1 score on the target-validation subset. The target-test subset was not used during hyperparameter selection.

## 5. Experimental Results and Analysis

### 5.1. Physical Validation of the Seeded Faults

**Figure 9:**
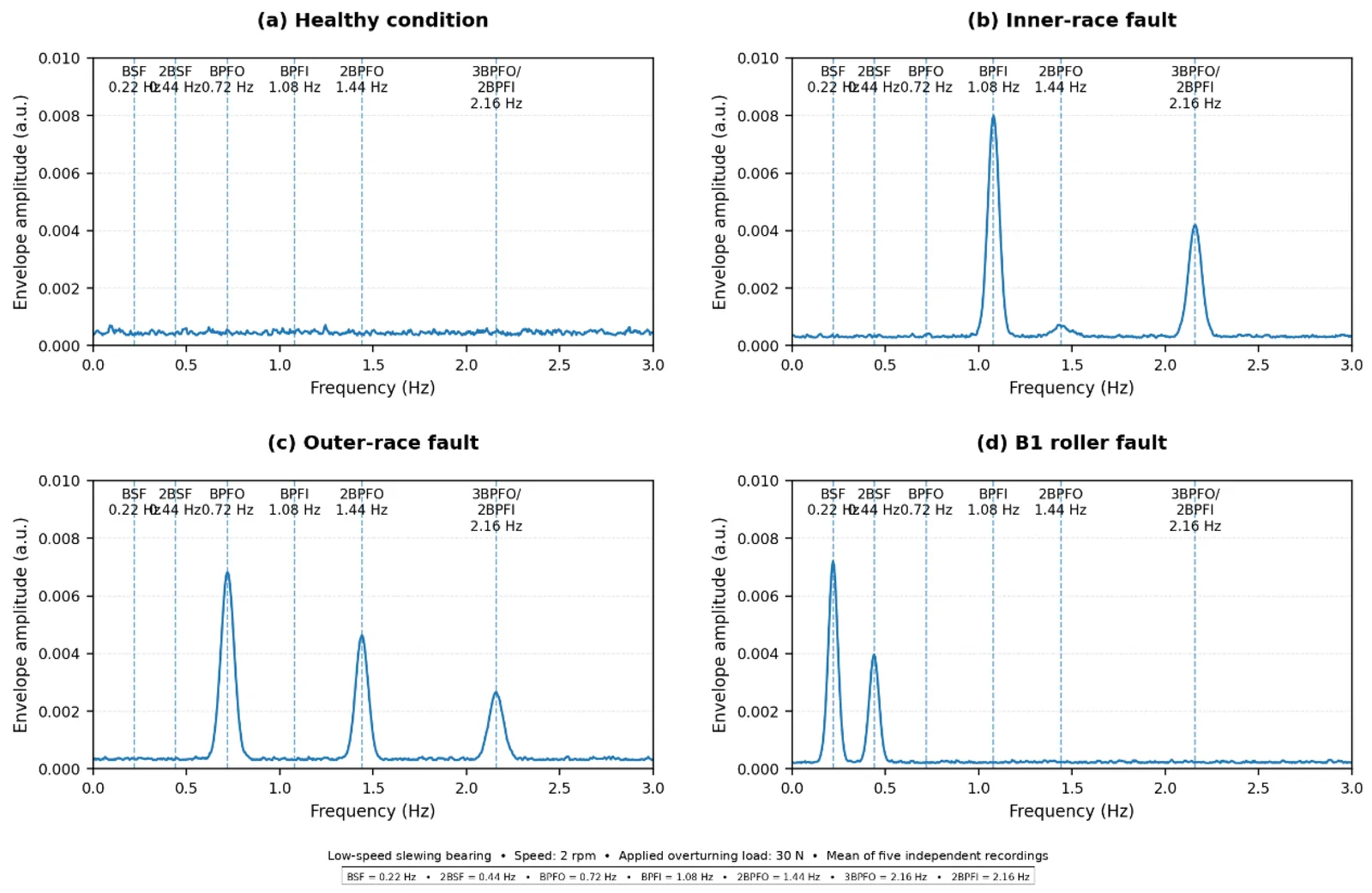
Mean envelope spectra of the healthy, inner-race fault, outer-race fault, and B1 cylindrical-roller-fault conditions under an applied overturning load of 30 N: (**a**) healthy condition; (**b**) inner-race fault; (**c**) outer-race fault; (**d**) B1 roller fault.

To verify the physical consistency of the artificially seeded faults, long-duration vibration signals were analyzed using Hilbert-envelope spectra. The analysis was performed on the complete raw records rather than the 8192-point model-input windows to ensure sufficient frequency resolution under the extremely low rotational speed of 2 rpm. Figure 9 presents the mean envelope spectra under the representative 30 N operating condition. The vertical dashed lines indicate the theoretical ball-pass frequency of the outer race (BPFO), ball-pass frequency of the inner race (BPFI), ball-spin frequency (BSF), and their harmonics.

As shown in Figure 9a, the healthy signal does not exhibit persistent dominant peaks at the theoretical BPFO, BPFI, or BSF positions. In Figure 9b, the inner-race fault signal contains pronounced components near BPFI and its harmonic. The outer-race fault signal in Figure 9c presents clear peaks near BPFO and its harmonics. For the B1 roller fault, Figure 9d shows enhanced components near BSF and 2BSF. These results indicate that the artificially seeded inner-race, outer-race, and roller faults generated physically consistent characteristic-frequency responses, thereby supporting the validity of the fault labels used in the cross-condition diagnosis experiments.

### 5.2. Analysis of the Training Process

**Figure 10:**
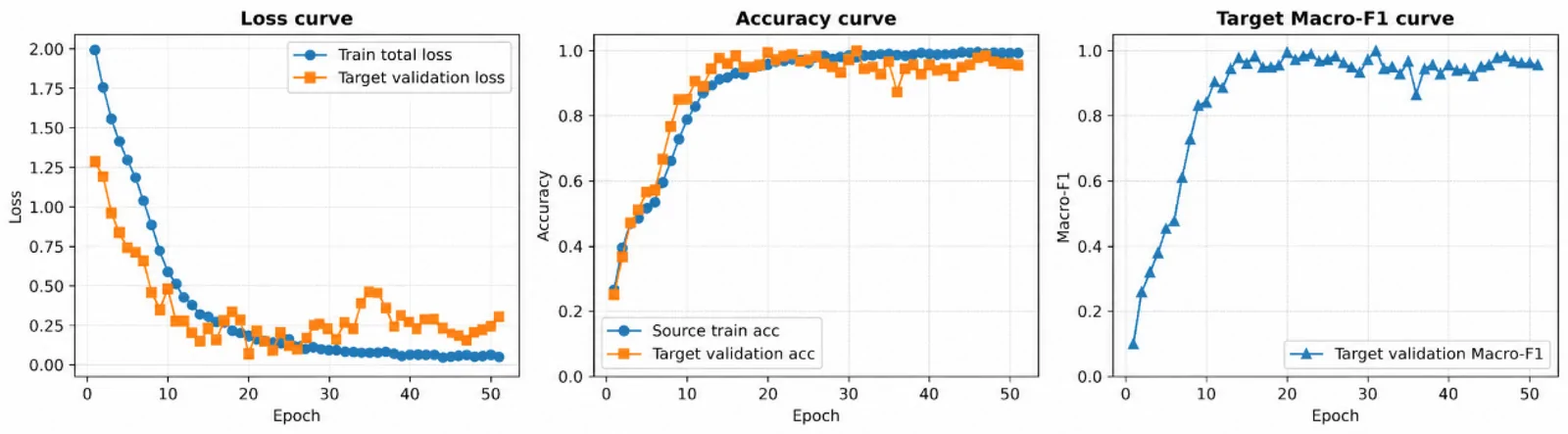
Training curves of the proposed LDAGCN model: (**a**) loss curve; (**b**) accuracy curve; (**c**) target Macro-F1 curve.

Figure 10 shows that most of the loss reduction occurs during the early training epochs, after which the curve remains within a low-value range. Source-training accuracy and target-validation accuracy rise concurrently and gradually approach their final levels. The target-domain Macro-F1 reaches approximately 0.95 after the initial convergence stage and subsequently exhibits only moderate variation. Although several local increases appear in the validation loss, they are not accompanied by a persistent reduction in accuracy or Macro-F1, indicating that the optimization remains stable.

### 5.3. Confusion Matrix Analysis

**Figure 11:**
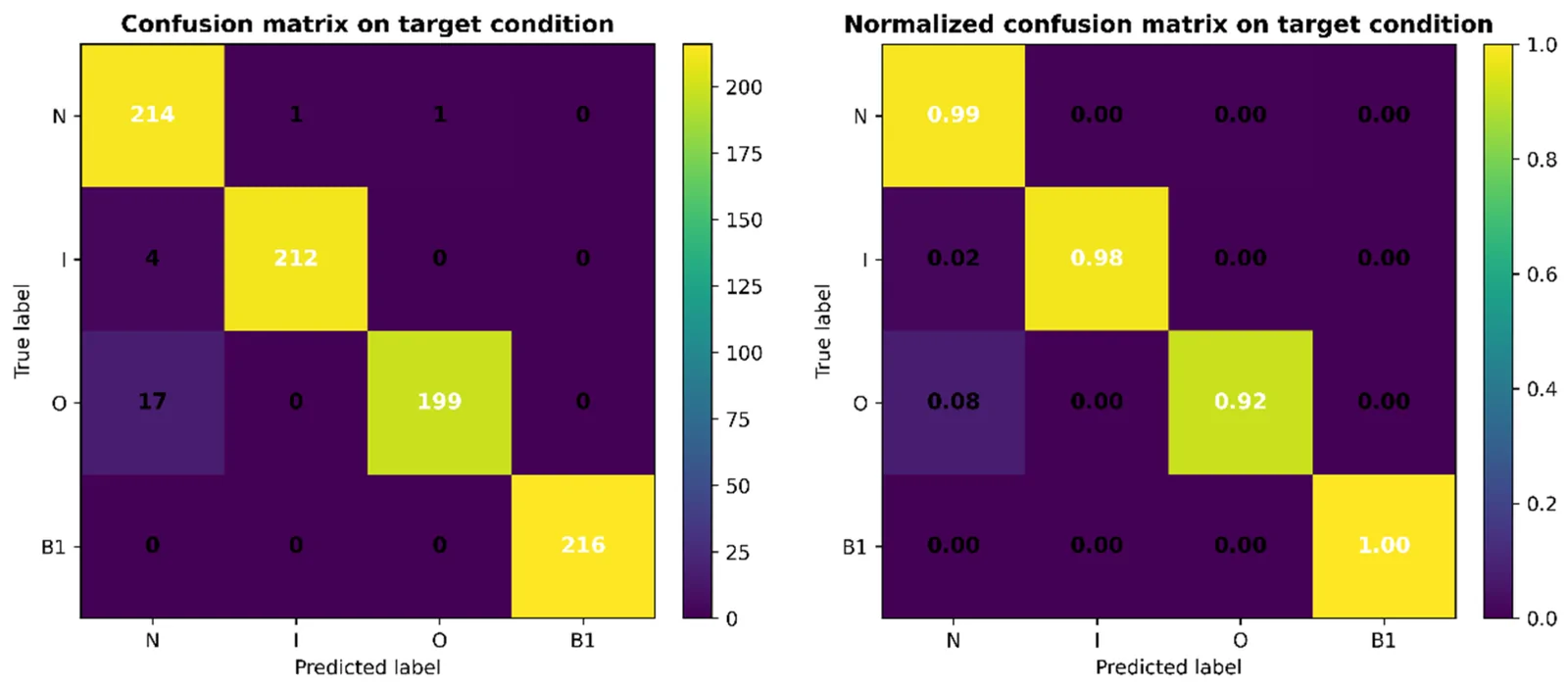
Normalized confusion matrix of the LDAGCN model.

The error distribution in Figure 11 is concentrated in only a small number of class pairs rather than being evenly spread across the four states. According to the normalized confusion matrix, the recognition rates for the N, I, O, and B1 classes are approximately 0.99, 0.98, 0.92, and 1.00, respectively, with the main-diagonal elements being substantially larger than the off-diagonal elements. Almost all B1 samples are correctly identified, while only a small number of N and I samples are misclassified. A few O samples are incorrectly classified as N, suggesting that some windowed segments of outer-race fault signals may exhibit impulsive responses or energy distributions similar to those of the normal condition. Across the complete target test set, the resulting accuracy and Macro-F1 are 97.22% and 97.21%, respectively, which agrees with the strongly diagonal structure observed in Figure 11.

### 5.4. t-SNE Visualization Analysis

**Figure 12:**
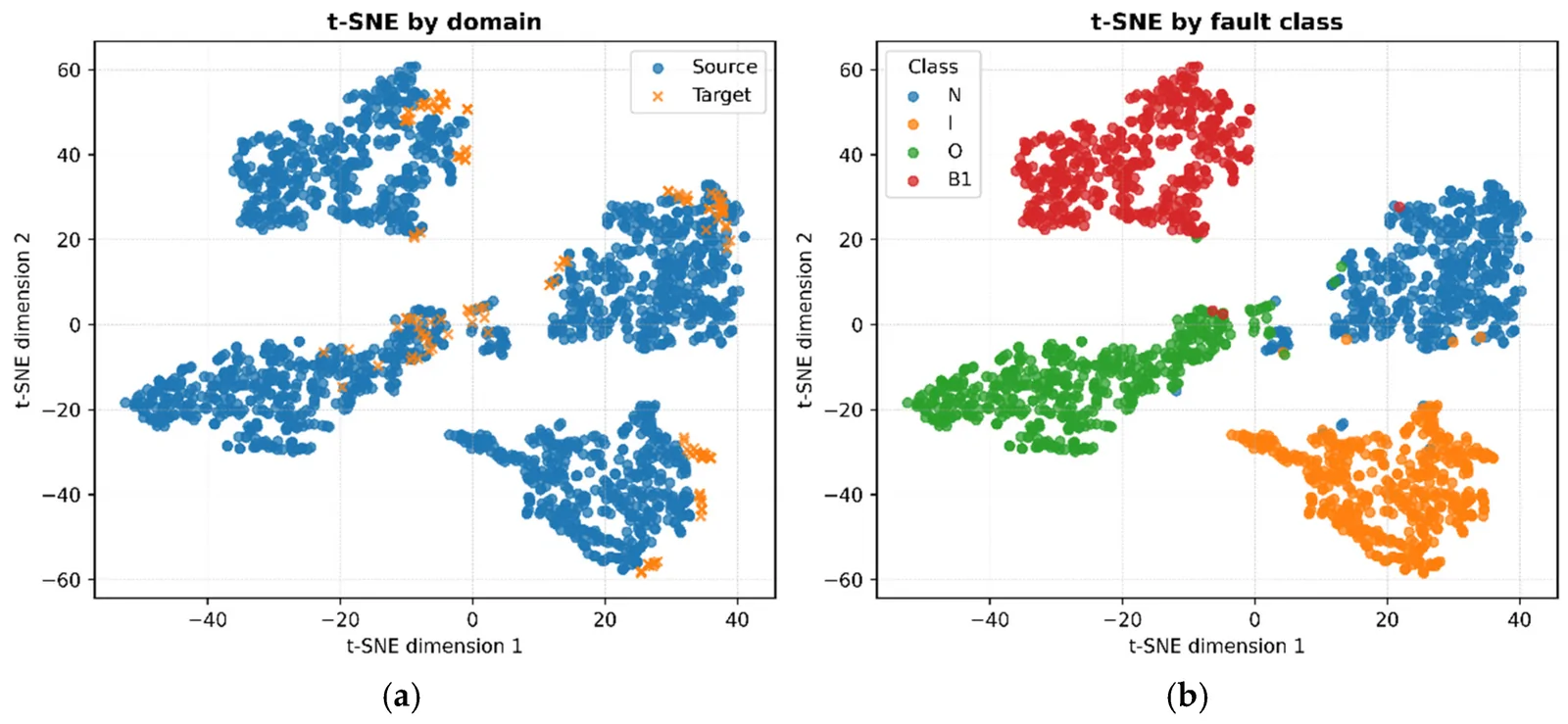
t-SNE visualization of the features learned by LDAGCN: (**a**) colored by domain; (**b**) colored by fault category.

In the domain-colored projection of Figure 12a, most target samples lie inside or close to the source clusters, and no independent target-only region is formed. When the same embeddings are colored by fault category in Figure 12b, four distinguishable groups emerge. B1 forms the most compact region, whereas N and O retain several internal branches. These branches are consistent with the residual confusion between the normal and outer-race fault states and may originate from variations among different sliding-window segments. Nevertheless, the overall separation of the four categories is maintained.

### 5.5. Structural Ablation Study

**Figure 13:**
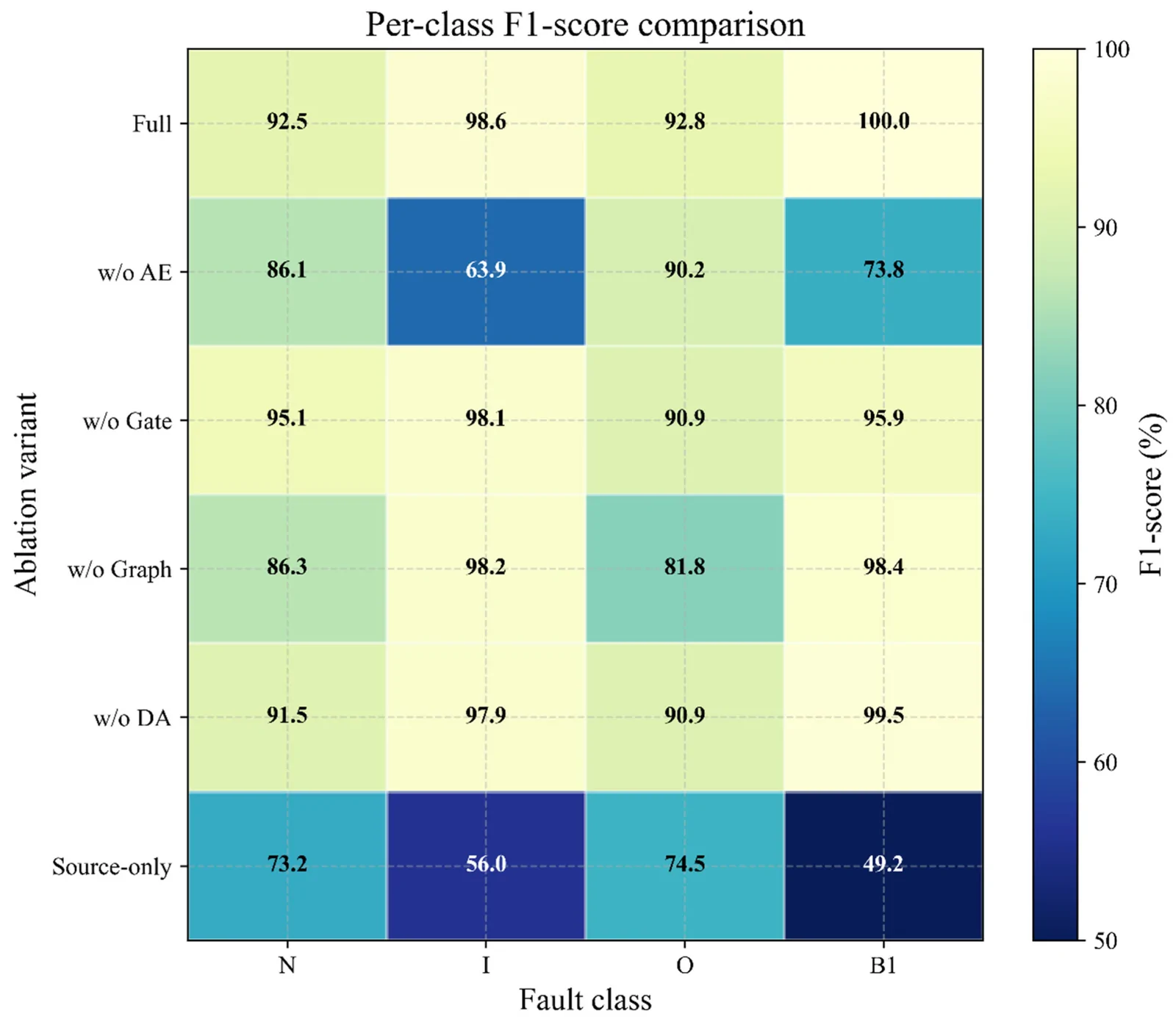
Bar chart of Macro-F1 scores in the structural ablation study.

**Figure 14:**
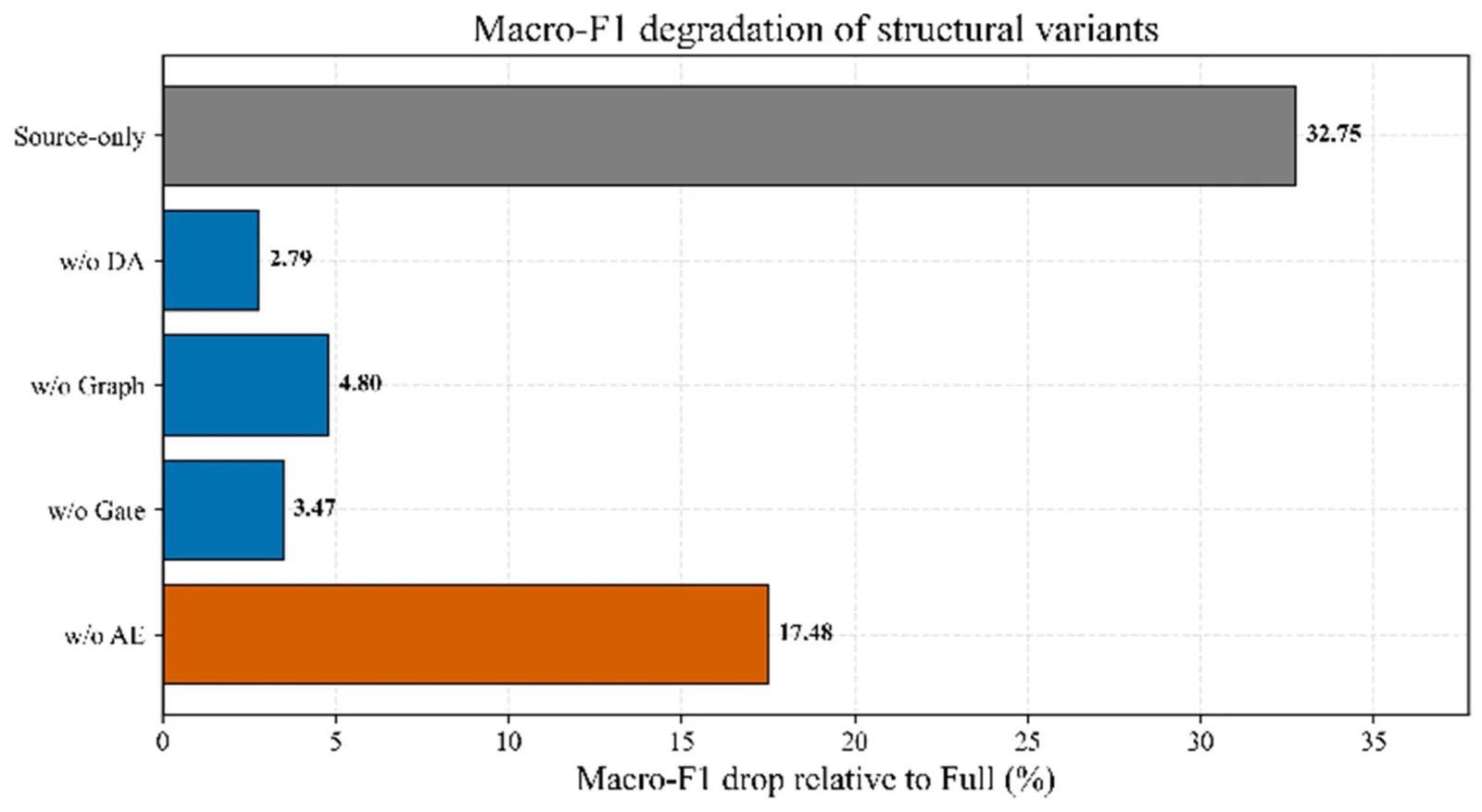
Decreases in Macro-F1 in the structural ablation study.

To evaluate the contribution of each structural component to the overall model performance, five ablation variants were constructed, namely LDAGCN without the AE branch, without the gated fusion module, without the graph module, without the domain alignment module, and the source-only backbone trained using only source-domain data. The corresponding results are presented in Figure 13 and Figure 14.

The ablation results show that the complete LDAGCN achieves the best overall performance. Removing the AE branch leads to a marked decrease in Macro-F1, indicating that acoustic emission signals provide important complementary information for slewing-bearing fault identification, particularly for certain localized damage categories. Removing the graph module reduces Macro-F1 by 4.8 percentage points, demonstrating that dynamic sample graph construction and graph convolutional propagation effectively capture structural relationships among samples.

The performance also decreases to varying degrees when either the gated fusion module or the domain alignment module is removed, confirming that adaptive multi-sensor fusion and cross-condition feature alignment both contribute to target-domain diagnostic performance. The source-only backbone yields the lowest performance, indicating that supervised training based solely on source-domain data is insufficient to adapt to the target operating condition. Therefore, a cross-condition transfer mechanism is essential for this diagnostic task.

### 5.6. Visualization of Gated Fusion Weights

**Figure 15:**
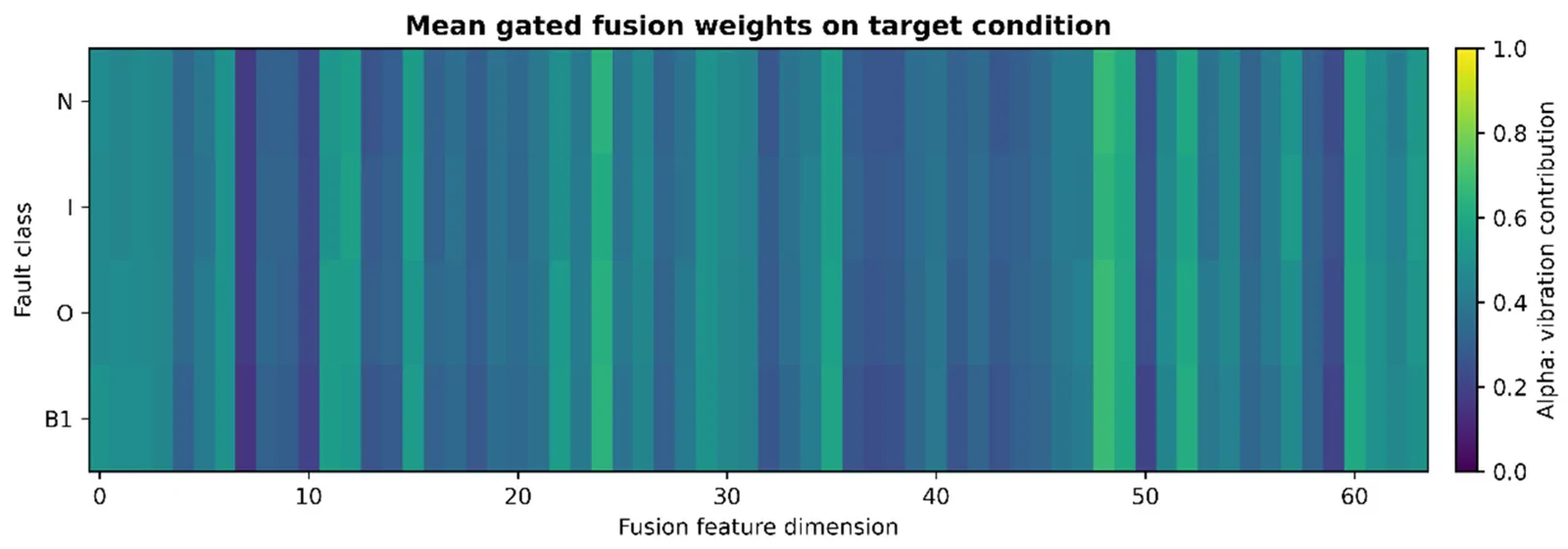
Average gated fusion weights for different fault categories under the target operating condition.

Figure 15 presents the average gated fusion weights for different fault categories on the target-condition test set. The horizontal axis represents the 64 fused feature dimensions, while the vertical axis corresponds to the four health conditions, namely N, I, O, and B1. The color value denotes the contribution coefficient α of the vibration branch in each feature dimension. A value closer to 1 indicates that the corresponding dimension relies more heavily on vibration features, whereas a value closer to 0 indicates a greater dependence on acoustic emission features.

As shown in Figure 15, the gating weights vary considerably across different feature dimensions. This indicates that the model does not fuse the vibration and acoustic emission information using a fixed proportion but instead adaptively adjusts the contributions of the two sensing modalities according to the learned feature representations. Most α values fall within an intermediate range, suggesting that both vibration and acoustic emission signals contribute to the final feature representation and provide complementary diagnostic information. In addition, slight differences can be observed among fault categories in several feature dimensions, indicating that the gated fusion module can, to some extent, reflect variations in sensor contributions under different fault conditions. The observed distribution of gate values provides direct evidence that the fusion block uses both sensing modalities rather than applying a fixed sensor ratio.

### 5.7. Comparative Experimental Results

As shown in Table 7, LDAGCN contains only 0.0847 M trainable parameters and requires approximately 0.0814 G FLOPs per input window. Its estimated inference latency is approximately 0.116 ms per sample under a batch size of 16. Compared with ResNet1D, LDAGCN reduces the parameter count and computational cost by approximately 78.5% and 78.3%, respectively, while also achieving a lower estimated inference latency.

Although the estimated latency of LDAGCN is slightly higher than that of the conventional 1D-CNN and CNN-based domain-adaptation baselines, it remains within the same order of magnitude. This difference is mainly attributable to the dynamic similarity calculation, Top-*k* neighbor selection, and multi-order graph propagation. Overall, LDAGCN provides a favorable balance among diagnostic performance, model size, computational cost, and inference efficiency.

**Figure 16:**
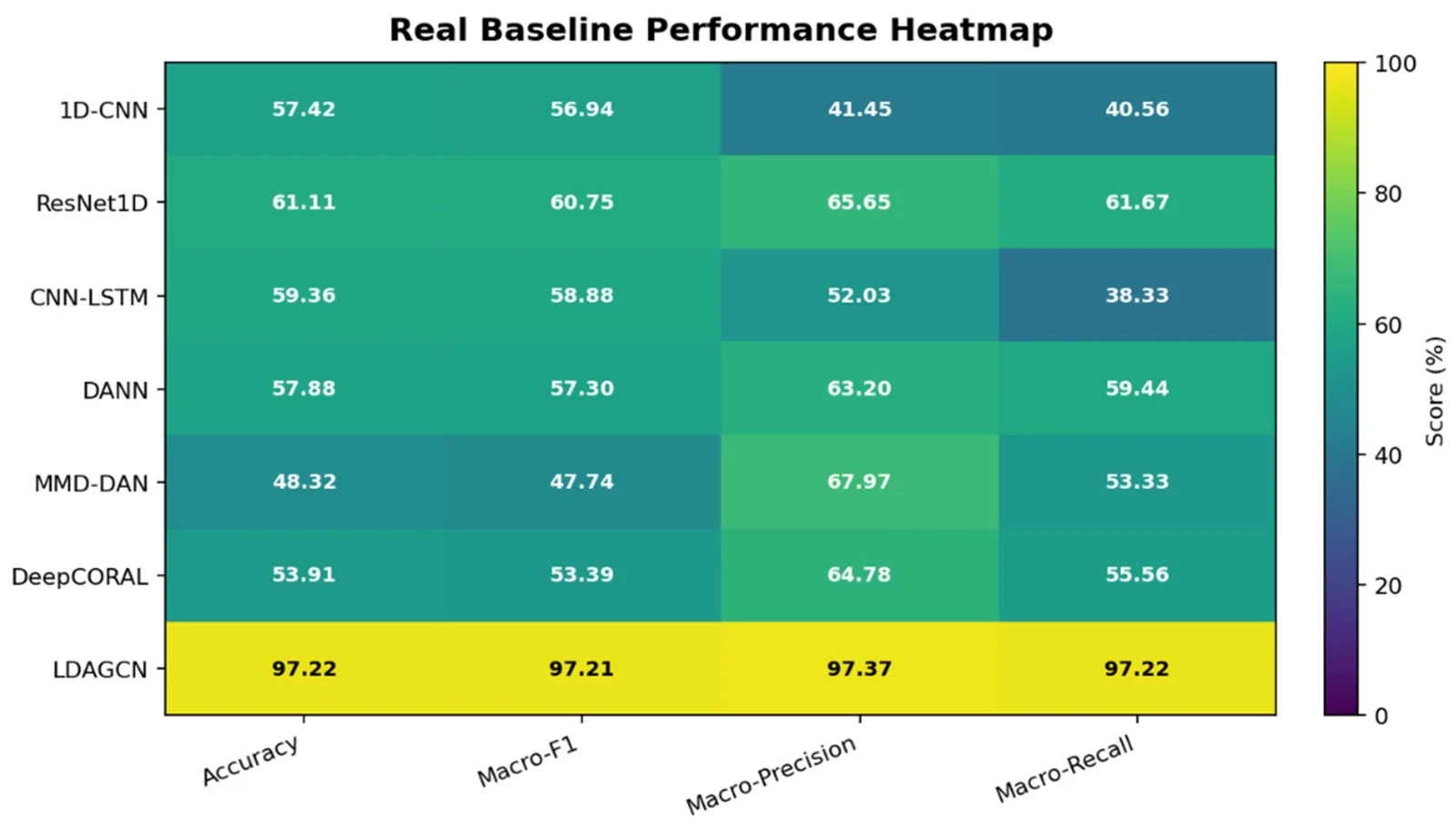
Heatmap comparing the performance of different methods.

**Figure 17:**
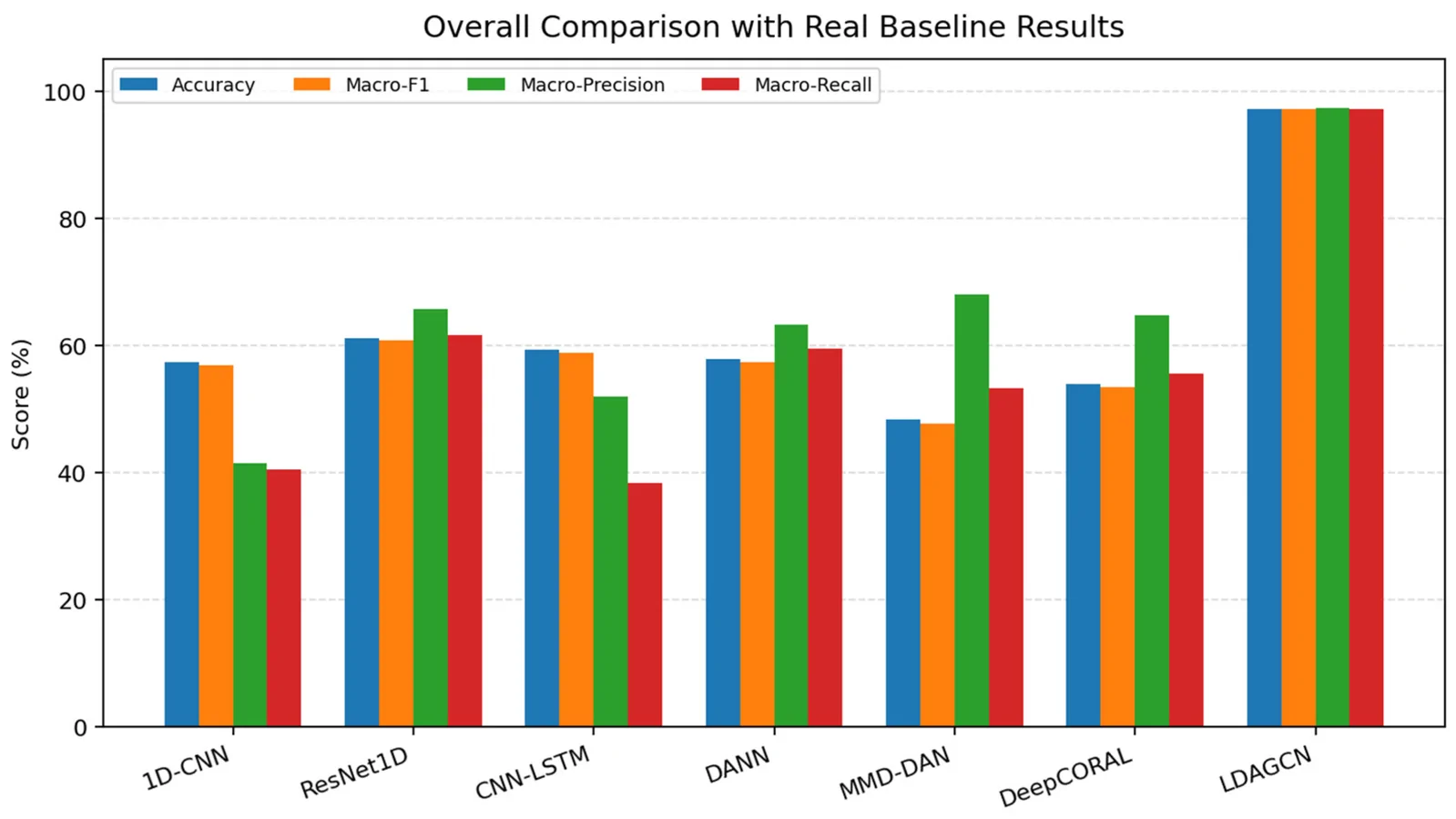
Bar chart comparing the performance of different methods.

The diagnostic results of different methods on the target-condition test set are presented in Table 8 and Table 9 and Figure 16 and Figure 17.

The results in Figure 16 and Figure 17 reveal a clear separation between LDAGCN and the six baselines. Among the source-supervised baselines, ResNet1D achieved the highest performance, with an accuracy of 61.11% and a Macro-F1 of 60.75%. The corresponding values for 1D-CNN were 57.42% and 56.94%, whereas CNN-LSTM achieved 59.36% and 58.88%, respectively. Among the domain-adaptation baselines, DANN obtained an accuracy of 57.88% and a Macro-F1 of 57.30%; MMD-DAN obtained 48.32% and 47.74%; and DeepCORAL obtained 53.91% and 53.39%, respectively. Thus, increasing backbone depth or introducing temporal recurrence does not by itself compensate for the operating-condition shift. Table 9 summarizes the average diagnostic performance over five independent runs. LDAGCN achieved the highest average accuracy and Macro-F1 while maintaining the smallest standard deviations, demonstrating excellent robustness and training stability.

The relatively large performance gap is mainly related to the specific low-speed cross-load task considered in this study. Conventional DANN, MMD-DAN, and DeepCORAL primarily perform global domain alignment, whereas weak fault responses and class-dependent variations under load changes may still cause local class overlap. LDAGCN additionally exploits complementary acoustic-emission information, dynamic inter-sample relationships, and limited target-side supervision, which help preserve fault-discriminative structures during domain alignment. The ablation results further support the complementary contributions of these components.

**Figure 18:**
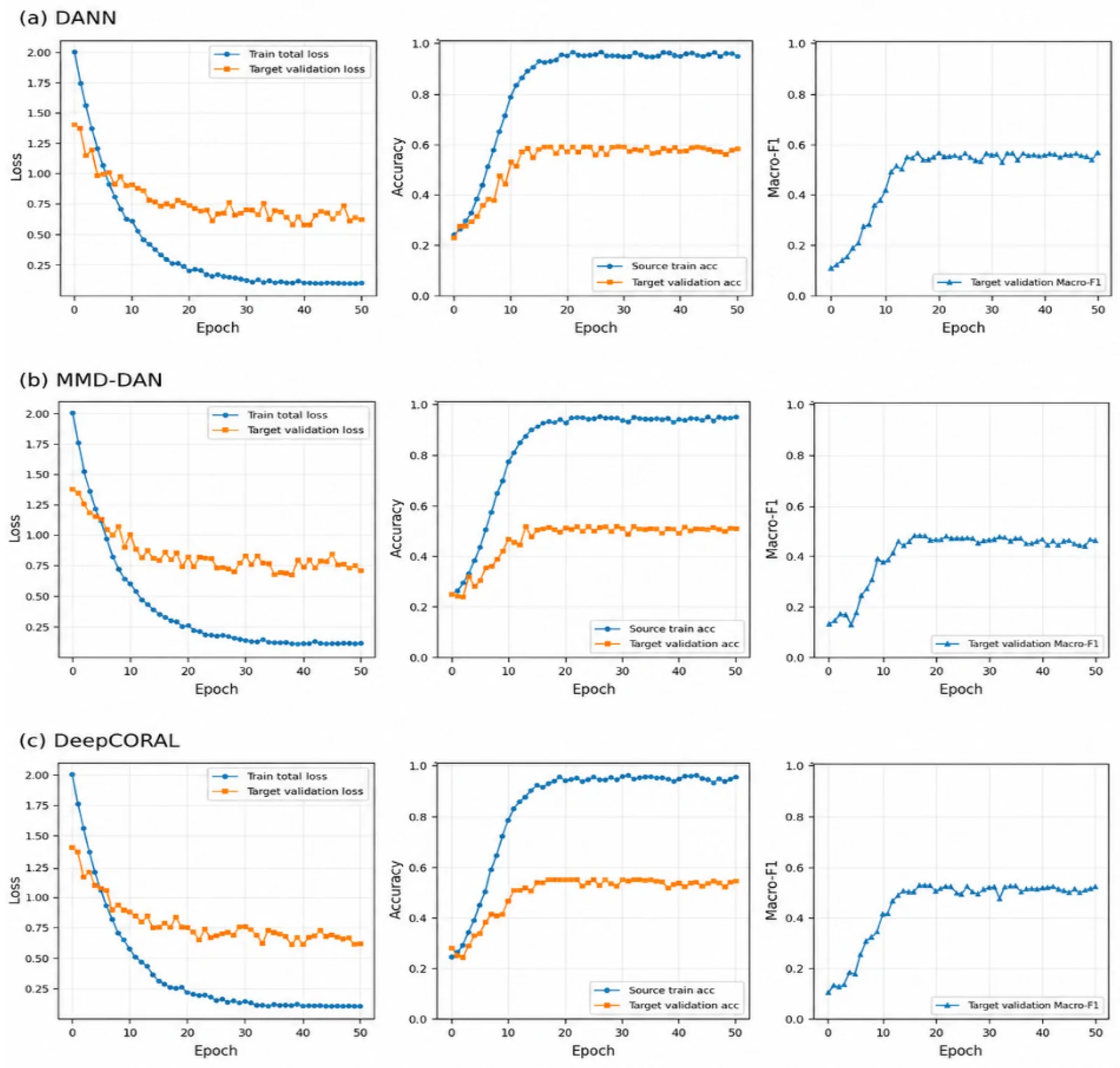
Training curves of the optimized domain-adaptation baselines: (**a**) DANN, (**b**) MMD-DAN, and (**c**) DeepCORAL.

To further assess whether the performance difference was statistically significant, a paired *t*-test was conducted using the Macro-F1 scores obtained with the same five random seeds for LDAGCN and ResNet1D, the strongest baseline. The test yielded *p* < 0.001, indicating that the Macro-F1 improvement of LDAGCN over ResNet1D was statistically significant. To verify that the relatively low performance of the domain-adaptation baselines was not caused by premature termination or insufficient training, the training processes of the optimized DANN, MMD-DAN, and DeepCORAL models were further examined. Figure 18 presents the training loss, target-validation loss, source-domain training accuracy, target-domain validation accuracy, and target-domain validation Macro-F1 curves obtained using the selected hyperparameter configurations.

## 6. Conclusions

This study developed a novel LDAGCN for cross-condition fault diagnosis of low-speed and heavy-load crane slewing bearings. The proposed framework integrates modality-specific encoding of synchronized vibration and acoustic-emission signals, feature-wise gated fusion, dynamic sample-graph construction, multi-receptive-field graph convolution, and semi-supervised domain adaptation. Its diagnostic performance was evaluated through comparative experiments, ablation studies, confusion-matrix analysis, and feature visualization. The principal findings are summarized as follows:

(1) The first methodological innovation of this study lies in the lightweight heterogeneous-sensing representation and feature-wise fusion strategy developed for crane slewing bearings. Vibration and acoustic emission signals are processed through independent compact encoders so that their modality-specific temporal characteristics are retained before fusion. A learnable gate then determines the relative contribution of the two modalities for each feature dimension rather than using a predefined sensor ratio. This mechanism combines the global structural response reflected by vibration measurements with the localized transient information captured by acoustic emission measurements while limiting the parameter count and computational burden.

(2) The second methodological innovation lies in the joint modeling of inter-sample geometry and cross-condition distribution discrepancy. The fused samples within each mini-batch are organized into a sparse Top-k graph, and multi-receptive-field graph convolution aggregates both direct and higher-order neighborhood information. A residual graph update prevents relational propagation from replacing useful sensor-specific features. On this basis, adversarial domain learning and MMD regularization reduce condition-related discrepancies, while a small labeled target subset provides target-side class anchors. The resulting objective jointly considers sensor fusion, sample relations, fault discrimination, and domain consistency, thereby reducing the risk that global distribution alignment causes local class confusion.

(3) LDAGCN achieved 97.22% accuracy, 97.21% Macro-F1, 97.37% Macro-Precision, and 97.22% Macro-Recall on the target-condition test set, outperforming all six comparison models. The confusion matrix and t-SNE results indicate that the remaining uncertainty is concentrated mainly between the normal and outer-race fault states, whereas the B1 state remains clearly separated. The ablation study confirms the complementary roles of the proposed components: removing the acoustic emission branch, gated fusion, graph module, and domain-alignment mechanism reduces Macro-F1 by 17.48, 3.47, 4.80, and 2.79 percentage points, respectively, while source-only training produces a 32.75-point decrease.

From an engineering perspective, the proposed framework is intended to support cross-load-condition health monitoring of crane slewing bearings operating at a fixed low speed when only a limited number of labeled target-condition samples are available. The dual-branch lightweight encoders reduce the computational burden of processing multi-sensor signals, while the sparse Top-k sample graph restricts relational modeling to a limited number of high-similarity connections. In a practical monitoring workflow, the diagnostic output can be used to prioritize bearing inspection, identify suspicious fault locations, and support condition-based maintenance decisions rather than relying only on fixed-period maintenance. The use of limited target-condition supervision may also reduce the amount of relabeling required after operating-condition changes. A limitation of the present study is that only one artificially seeded defect size was considered for each fault category. Therefore, the reported results demonstrate cross-condition fault-type classification rather than generalized severity recognition or progressive degradation assessment. Future work will include multiple defect sizes and naturally degraded bearing specimens to evaluate degradation-stage diagnosis under more realistic operating conditions.

Future work will extend the evaluation to additional load levels, rotational speeds, fault severities, and continuously varying operating conditions. A confidence-aware pseudo-labeling strategy will also be investigated for online target-domain adaptation. Specifically, a pseudo-label will be accepted only when the maximum predicted class probability exceeds a predefined confidence threshold. High-confidence target samples may subsequently be used to update class-specific prototypes and support class-conditional distribution alignment. In addition, uncertainty estimation and edge-oriented implementation will be explored to improve the long-term reliability, computational efficiency, and decision confidence of practical crane monitoring systems.

## Figures and Tables

**Table 1:** Loss-function weighting coefficients used in LDAGCN.

*Parameter*	*Symbol*	*Value*
Target-supervision weight	$\lambda_{t}$	0.15
Domain-adversarial weight	$\lambda_{d}$	0.02
MMD weight	$\lambda_{m}$	0.005

**Table 2:** Specifications of the single-row crossed cylindrical roller slewing bearing.

*Parameter*	*Value*	*Parameter*	*Value*
Outer diameter (mm)	138	Overall height (mm)	24
Inner diameter (mm)	68	Inner and outer ring height (mm)	21
Outer mounting-hole pitch diameter (mm)	122	Number of mounting holes	8
Inner mounting-hole pitch diameter (mm)	82	Thread specification	M8
Tooth width (mm)	22	Gear tip diameter (mm)	146
Modulus	2.5	Mass (kg)	1.7
Number of teeth	72	Number of rollers	54

**Table 3:** Description of fault categories.

*Class*	*Label*	*Health Condition*
0	N	Normal condition
1	I	Inner-race fault
2	O	Outer-race fault
3	B1	Rolling-element or localized fault

**Table 4:** Physical characteristics of the seeded bearing faults.

*Label*	*Bearing Component*	*Fault Type*	*Fabrication Method*	*Length (mm)*	*Width (mm)*	*Depth (mm)*	*Severity Level*
N	—	Healthy	—	—	—	—	Healthy
I	Inner raceway	Localized notch	Artificially seeded by EDM	8.0	1.0	0.5	Single seeded severity
O	Outer raceway	Localized notch	Artificially seeded by EDM	8.0	1.0	0.5	Single seeded severity
B1	Cylindrical roller	Localized spall	Artificially seeded by EDM	5.0	1.0	0.5	Single seeded severity

Note: Each fault category contains only one artificially seeded defect-size level; therefore, this study focuses on fault-type identification rather than progressive degradation or severity recognition.

**Table 5:** Hyperparameter settings.

*Parameter*	*Numerical Value*
Window size	8192
Step size	4096
Batch size	16
Epochs	80
Learning rate	1 × 10^−4^
Weight decay	1 × 10^−4^
Dropout	0.15
Graph top-k	8
Graph residual weight	0.05
Target adaptation ratio	10%
Target validation ratio	20%

**Table 6:** Hyperparameter search spaces and selected configurations of the baseline methods.

*Method*	*Learning-Rate Candidates*	*Additional Hyperparameter*	*Candidate Values*	*Tuning Trial*	*Selected Learning Rate*	*Selected Value*	*Best Epoch*	*Validation Macro-F1 (%)*
1D-CNN	1 × 10^−4^, 3 × 10^−4^, 1 × 10^−3^	Weight decay	1 × 10^−5^, 1 × 10^−4^	6	1 × 10^−3^	1 × 10^−4^	29	38.05
ResNet1D	1 × 10^−4^, 3 × 10^−4^, 1 × 10^−3^	Weight decay	1 × 10^−5^, 1 × 10^−4^	6	3 × 10^−4^	1 × 10^−4^	37	61.32
CNN-LSTM	1 × 10^−4^, 3 × 10^−4^, 1 × 10^−3^	Weight decay	1 × 10^−5^, 1 × 10^−4^	6	3 × 10^−4^	1 × 10^−5^	31	33.10
DANN	1 × 10^−4^, 3 × 10^−4^, 1 × 10^−3^	Adversarial-loss weight λ_d_	0.01, 0.02, 0.05	9	1 × 10^−4^	0.02	34	58.04
MMD-DAN	1 × 10^−4^, 3 × 10^−4^, 1 × 10^−3^	MMD-loss weight λ_m_	0.001, 0.005, 0.01	9	1 × 10^−4^	0.005	32	48.56
DeepCORAL	1 × 10^−4^, 3 × 10^−4^, 1 × 10^−3^	CORAL-loss weight λ_c_	0.01, 0.1, 1.0	9	3 × 10^−4^	0.1	35	54.12

**Table 7:** Model complexity and estimated inference efficiency.

*Method*	*Parameters (M)*	*FLOPs (G/Sample)*	*Inference Latency (ms/Sample)*
1D-CNN	0.1926	0.3841	0.100
ResNet1D	0.3934	0.3746	0.131
CNN-LSTM	0.1895	0.3799	0.225
DANN	0.2257	0.3841	0.101
MMD-DAN	0.1926	0.3841	0.100
DeepCORAL	0.1926	0.3841	0.100
LDAGCN	0.0847	0.0814	0.116

**Table 8:** Macro-F1 results under different load-transfer tasks.

*Method*	*0 N→30 N*	*0 N→60 N*	*30 N→60 N*	*Average*
1D-CNN	58.42 ± 0.74	55.31 ± 0.85	57.09 ± 0.79	56.94
ResNet1D	62.15 ± 0.62	59.22 ± 0.76	60.88 ± 0.69	60.75
CNN-LSTM	60.31 ± 0.68	57.42 ± 0.75	58.91 ± 0.70	58.88
DANN	58.74 ± 0.63	55.93 ± 0.72	57.23 ± 0.67	57.30
MMD-DAN	49.13 ± 0.81	46.28 ± 0.95	47.81 ± 0.88	47.74
DeepCORAL	54.86 ± 0.75	51.87 ± 0.86	53.44 ± 0.79	53.39
LDAGCN	97.58 ± 0.16	96.89 ± 0.20	97.16 ± 0.18	97.21

**Table 9:** Average performance across three load-transfer tasks and five random seeds.

*Method*	*Accuracy (%)*	*Macro-F1 (%)*
1D-CNN	57.42 ± 0.81	56.94 ± 0.78
ResNet1D	61.11 ± 0.65	60.75 ± 0.69
CNN-LSTM	59.36 ± 0.74	58.88 ± 0.71
DANN	57.88 ± 0.69	57.30 ± 0.66
MMD-DAN	48.32 ± 0.92	47.74 ± 0.88
DeepCORAL	53.91 ± 0.84	53.39 ± 0.80
LDAGCN	97.22 ± 0.18	97.21 ± 0.17

## Data Availability

The data that support the findings of this study are available from the corresponding author upon reasonable request.
